# Long-term visual localization in dynamic benthic environments: the SEALOC dataset, footprint-based ground truth, and visual place recognition benchmark

**DOI:** 10.3389/frobt.2026.1821019

**Published:** 2026-06-08

**Authors:** Martin Kvisvik Larsen, Oscar Pizarro

**Affiliations:** Department of Marine Technology, NTNU, Trondheim, Norway

**Keywords:** autonomous underwater vehicle, benthic environments, dataset, ground truth, visual localization, visual place recognition

## Abstract

Long-term visual localization has the potential to reduce cost and improve mapping quality in optical benthic monitoring with autonomous underwater vehicles Despite this potential, long-term visual localization in benthic environments remains understudied, primarily due to the lack of curated datasets for benchmarking. Moreover, limited georeferencing accuracy and image footprints necessitate precise geometric information for accurate ground-truthing. In this work, we address these gaps by presenting SEALOC, a curated dataset for long-term visual localization in benthic environments, and a novel method to ground-truth visual localization results for near-nadir underwater imagery. Our dataset comprises georeferenced AUV imagery from five benthic reference sites, revisited over periods up to 6 years, and includes raw and color-corrected stereo imagery, camera calibrations, and sub-decimeter registered camera poses. To our knowledge, this is the first curated underwater dataset for long-term visual localization spanning multiple sites and photic-zone habitats. Our ground-truthing method estimates 3D seafloor image footprints and links camera views with overlapping footprints, ensuring that ground-truth links reflect shared visual content. Building on this dataset and ground truth, we benchmark eight state-of-the-art visual place recognition (VPR) methods and find that Recall@K is significantly lower on our dataset than on established terrestrial and underwater benchmarks. Finally, we compare our footprint-based ground truth to a traditional location-based ground truth and show that distance-threshold ground-truthing can overestimate visual place recognition Recall@K at sites with rugged terrain and altitude variations. Together, the curated dataset, ground-truthing method, and VPR benchmark provide a stepping stone for advancing long-term visual localization in dynamic benthic environments.

## 1 Introduction

Optical monitoring of benthic habitats using robotic platforms is changing marine ecological research by providing high-resolution, repeatable imagery over broad areas and depths where human access is limited (Misiuk and Brown, 2024). Cameras mounted on autonomous underwater vehicles (AUVs) or remotely operated vehicles (ROVs) enable detailed, non-destructive mapping of species and habitat structures, supporting quantitative and systematic seafloor assessments that were previously unattainable.

Due to the unavailability of global navigation satellite system (GNSS), underwater robotic platforms often depend on acoustic positioning systems (APSs) for navigation and image georeferencing (Wu et al., 2019). However, deploying APSs involves substantial costs and logistical overhead, requiring auxiliary infrastructure such as topside and subsea transducers, a support vessel for tracking, and specialized personnel for setup and calibration. In addition, APSs are highly sensitive to calibration and alignment errors (Chen, 2008; Jinwu et al., 2018; Zhu et al., 2020). For benthic repeat surveys conducted months or years apart, calibrations can be invalidated due to sensor drift or misalignment after hardware reinstallation. Consequently, image georeferencing accuracy across visits to the same benthic location is typically limited to several meters (Bryson et al., 2013a; Larsen et al., 2023).

Long-term visual localization provides a means for underwater robots to estimate their position by recognizing distinctive features of the seafloor observed during previous surveys. In this approach, the visual structure of the benthic environment itself serves as a natural reference for vision-aided navigation (VAN) (Mahon et al., 2008; Eustice et al., 2008; Zhang et al., 2023), reducing the need for continuous external tracking and costly APS infrastructure. Beyond logistical benefits, visual localization also enhances the spatial quality of benthic imaging datasets by enabling precise relocalization within previously mapped areas, ensuring consistent coverage and sufficient image overlap across revisits. This supports centimeter-scale registration of imagery between surveys, facilitating detection of small-scale habitat changes such as species turnover or morphological alterations. Temporally registered imagery has, for instance, been shown to enable attribution of structural habitat changes to specific taxonomic groups (Ferrari et al., 2016).

Despite its promise, long-term visual localization in benthic environments remains challenging. Images captured by underwater robots are degraded by strong light attenuation, backscatter, and non-uniform illumination, all of which diminish image quality and limit the effective sensing range and image footprint (Song et al., 2022). Additionally, the temporal dynamics of benthic habitats, including among other the growth, decay, and death of sessile organisms, sediment disturbances, and collapse of structural features, further challenge the robustness of established visual localization methods when applied to imagery collected over months or years (Bryson et al., 2013a).

Advances in long-term visual localization research for terrestrial environments have largely been driven by the availability of curated datasets that support systematic method development and evaluation (Toft et al., 2022). In contrast, similar datasets from underwater environments remain rare. To date, the only curated underwater dataset featuring multiple revisits over extended periods is the Eiffel Tower dataset (Boittiaux et al., 2023), which consists of ROV imagery of a hydrothermal vent in the Lucky Strike vent field on the Mid-Atlantic Ridge at ∼1,700 m depth. The dataset consists of imagery collected during visits in 2015, 2016, 2018, and 2020, and covers an area of ∼ 40×40 meters at a spatial resolution of ∼2.4 mm/pixel.

In this work, we present SEALOC, a curated imaging AUV dataset designed for long-term visual localization research in benthic environments. The dataset was curated through automated detection and manual selection of benthic reference sites covering diverse seafloor types, with high imagery overlap across visits and suitability for robust 3D reconstruction, excluding sites with insufficient georeferencing quality or highly dynamic biological cover such as kelp. For each selected site, image color correction was applied to ensure color consistent imagery across visits, and geometric reconstruction and registration were used to obtain camera calibrations and sub-decimeter registered camera poses. The dataset addresses the lack of resources capturing the natural temporal variability of photic-zone habitats, providing a basis for developing robust methodologies for underwater visual localization. The dataset consists of repeat surveys at five benthic reference sites located at depths between ∼18 and ∼45 m. Each site spans approximately 35×35 meters and is mapped at a spatial resolution of ∼1.3 mm/pixel. For each site, we provide raw and color-corrected stereo imagery, along with camera calibrations and geometrically registered camera poses.

Unlike the Eiffel Tower dataset (Boittiaux et al., 2023), which captures a hydrothermal vent at ∼1,600 m depth, the SEALOC dataset covers photic-zone environments that experience strong temporal dynamics driven by ambient conditions such as light availability and water temperature, and episodic events such as extreme storms and marine heatwaves (Wong et al., 2023; Perkins et al., 2025). These dynamics manifest as visual and structural changes that challenge long-term visual localization. Biologically, rapid growth and succession of sessile organisms and episodic bleaching events alter community composition, texture, and color over months to years, challenging the ability of visual localization methods to match scenes across revisit intervals. Storms can cause sediment disturbances that bury previously visible features and reorganize rock-sand interfaces, while variability in ambient light and water optical properties shifts the apparent color and contrast of the scene between visits. In contrast, hydrothermal vent communities at the Mid-Atlantic Ridge are considered stable over decadal timescales in the absence of major eruption events (Glover et al., 2010). These contrasting conditions make photic-zone habitats a more challenging testbed for long-term visual localization.

Building on this dataset, we present a benchmark of eight state of the art (SOTA) visual place recognition (VPR) models to evaluate their performance in dynamic benthic environments. Evaluation of visual localization methods for near-nadir (i.e., “down-looking”) AUV imagery brings unique challenges, as traditional ground-truthing based on location proximity thresholds may be inadequate where the camera’s sensing range is similar to the relief of the seafloor. Previous studies (Larsen et al., 2023; Gorry et al., 2025) typically classified true localizations by requiring the location of the query and database images to be within a fixed distance. However, empirical evidence indicates that this approach can lead to incorrect assessments when the sensing range changes throughout the dataset, either due to sudden changes in seafloor relief or variations in vehicle altitude (Larsen et al., 2023). To address these limitations, we propose a ground-truthing method that leverages range data to estimate the 3D coordinate of the image corners onto the seafloor, allowing a geometric computation of each image’s footprint. True localizations are then classified by footprint overlap between the query and database images, ensuring that positive matches are classified based on common visual content, rather than location proximity alone.

Collectively, our curated dataset, ground-truthing approach, and comprehensive VPR benchmark are intended to support and accelerate the development of models and methods for long-term visual localization in dynamic benthic environments. Our main contributions are:

- The SEALOC dataset, a curated dataset of AUV imagery for long-term visual localization in benthic environments, providing geometrically registered camera poses, camera calibrations, raw and color-corrected stereo imagery. The dataset is the first curated dataset for this purpose that covers habitats from the photic zone and includes five benthic reference sites with diverse seafloor types revisited over periods up to 6 years.
- A ground-truthing approach for visual localization results for near-nadir AUV imagery, using overlapping image footprints to classify correct localization and eliminating the need for location proximity thresholds.
- A comprehensive benchmark of established VPR methods assessed on the new dataset, advancing the development and evaluation of robust visual localization techniques for repeatable optical mapping of benthic habitats.
- We show that VPR performance metrics are affected by the choice of ground truth definition by comparing our footprint-based ground truth to a location-based ground truth. We show that our footprint-based ground truth yields consistent results across sites, and that the location-based ground truth overestimates model performance for sites with rugged terrain or visits with large altitude variations.

The rest of the paper is structured as follows. In Section 2.1, we describe the data sources for the SEALOC dataset. Section 2.2, Section 2.3, and Section 2.4 detail the data curation methodology, including data selection, image color correction, and geometric reconstruction and registration to obtain camera calibrations and precise relative camera poses across visits. Section 2.5 presents our methodological contribution for estimating image footprints and identifying overlapping footprints to establish ground-truth correspondences for visual localization. Section 2.6 introduces the VPR benchmark, including the selected VPR models and evaluation protocol. Section 3 presents results from the dataset curation, footprint-based ground truth, and VPR benchmarking. Finally, Section 4 discusses these findings, examines implications for long-term visual localization in benthic environments, and outlines directions for further work.

## 2 Methods

### 2.1 Data source

Long-term, georeferenced optical monitoring datasets of the seafloor are rare, particularly those that repeatedly image fixed reference sites at high spatial resolution over multiple years. Australia’s Integrated Marine Observing System (IMOS) addresses this gap through a national benthic imaging program that acquires optical AUVs dataset along the continental shelf, targeting reef habitats and other structurally complex seafloor types that are sensitive to environmental change (Williams et al., 2012). Within this program, the IMOS AUV Facility operates a network of benthic reference sites that are revisited on multi-year timescales, following a nested sampling design that combines broad, sparse grids with dense “mow-the-lawn” grids on the order of 25×25 m to support repeat mapping and quantitative change detection (Pizarro et al., 2013). The imagery in the SEALOC dataset was collected by AUV Sirius, the primary benthic imaging platform of the IMOS AUV Facility (Williams et al., 2012). AUV Sirius is a mid-size SeaBED-class vehicle designed for low-speed, near-bottom surveys, with passive stability in pitch and roll to enable consistent, near-nadir optical imaging (Pizarro et al., 2013). The vehicle executes pre-planned lawnmower patterns that provide dense, overlapping coverage over reference grids, using a near-nadir stereo camera rig with artificial light sources for high-resolution imaging. Its navigation system fuses Doppler velocity log (DVL), attitude, depth, acoustic positioning, and visual simultaneous localization and mapping (SLAM) (Mahon et al., 2008) to produce self-consistent trajectories and precisely georeferenced imagery suitable for 3D reconstruction and repeat surveys of permanent reference sites. A more detailed overview of AUV Sirius’ sensor suite and the technical specifications for individual sensors are provided in Supplementary Materials.

### 2.2 Data selection

The SEALOC dataset is derived from the archive of benthic imagery collected by AUV Sirius across multiple IMOS campaigns and deployments and made accessible through the IMOS–UMI Squidle+ web portal (Friedman et al., 2025). From this archive, we sought regions that (i) were mapped with dense, overlapping coverage during individual visits and (ii) were revisited at least three times, providing overlapping views of the same seafloor patches over time. To automatically detect such regions, the clustering-based method of Larsen et al. (2023) was applied to the georeferenced image locations on Squidle+, yielding spatial clusters that correspond to densely mapped areas and serve as candidate benthic reference sites. The raw data for deployments containing these clusters was then retrieved from internal Australian Centre of Field Robotics (ACFR) data servers.

**FIGURE 1:**
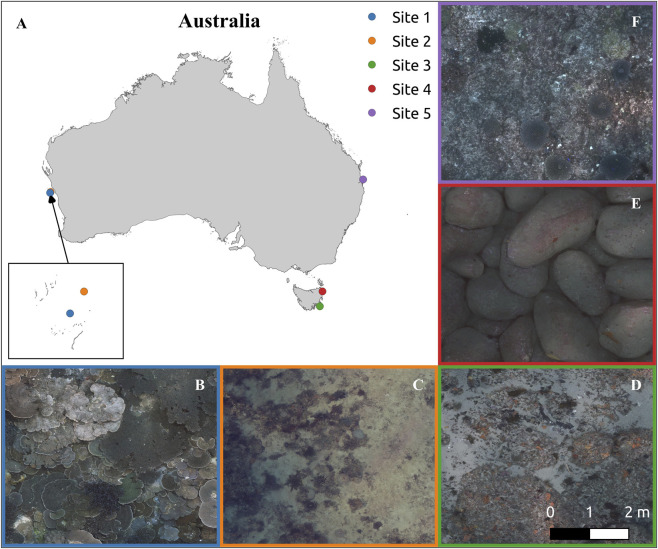
Map with overview of the benthic reference sites in the SEALOC dataset. **(A)** Map of Australia and the geographic location of each site. **(B–F)** Orthomosaics showing seafloor patches with characteristics representative for the following benthic reference sites; **(B)** Site 1, **(C)** Site 2, **(D)** Site 3, **(E)** Site 4, and **(F)** Site 5. The orthomosaic patches are rendered at the same spatial scale, indicated by the scalebar in the lower right corner. Base map data ⓒ Commonwealth of Australia (Australian Bureau of Statistics) 2021, ASGS Edition 3 digital boundaries, used under CC BY 4.0 (Australian Bureau of Statistics, 2021).

From the set of candidate clusters, five reference sites were selected to constitute the final benchmark dataset. Each site required at least three visits from different years with overlapping coverage, sufficient image counts and georeferencing quality to support robust 3D reconstruction. Sites dominated by highly dynamic cover such as kelp were excluded due to reconstruction quality. The selected sites span depths of approximately 18–45 m and encompass diverse habitat types, including sparse and dense coral reefs, soft sediment bottom, rock reefs, and boulder reefs. Figure 1 summarizes the spatial distribution and representative orthomosaic patches for each site, and Table 1 provides an overview of their key properties.

**TABLE 1:** Overview of the benthic reference sites in the SEALOC dataset.

Site	Geohash	Visit years	Depth range [m]	Image count	Site description
Site 1	QDCH0FTQ	2010, 2011, 2012, 2013	17–20	17,794	Dense coral reef
Site 2	QDCHDMY1	2011, 2012, 2013, 2017	27–35	20,944	Dense coral reef and soft sediment
Site 3	R23685BC	2010, 2012, 2014	40–43	15,146	Rock reef with corals and sponges and sandy substrate
Site 4	R29MRD5H	2009, 2011, 2013	25–36	21,362	Boulder reef
Site 5	R7JJSKXQ	2010, 2012, 2013	18–21	19,444	Rock reef with corals and sponges

### 2.3 Image color correction

Imagery captured by underwater robotic platforms is affected by wavelength-dependent attenuation, backscatter, and non-uniform artificial illumination, which together cause strong color distortions and spatial brightness variations. To improve the color consistency of the imagery in the SEALOC dataset, we apply the multi-image gray-world algorithm (Bryson et al., 2013b), which is designed to correct spatial color variations caused by lens vignetting and non-uniform lighting. In contrast to the conventional gray-world assumption over a single image (Buchsbaum, 1980), this method estimates per-pixel statistics over multiple images from the same camera, and then applies a linear per-pixel correction that equalizes the mean and variance of the color channels across the image dataset.

**FIGURE 2:**
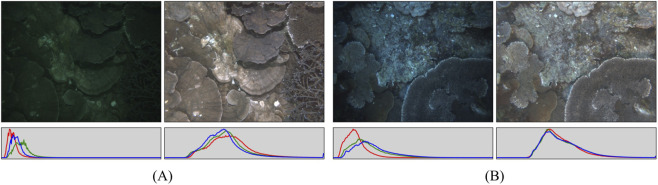
**(A)** Raw (left) and color corrected (right) image from the 2010 visit to Site 1. **(B)** Raw (left) and color corrected (right) image from the 2013 visit to Site 1.

For each camera and visit, we estimate these correction factors from the raw images and apply the resulting linear transform to all images for that camera and visit, yielding color-corrected imagery with more uniform illumination and improved color balance. Images are normalized to the $\left[0,1\right]$ intensity range before correction, and we set the desired mean and standard deviation of the corrected intensities to $\mu_{y}\left(\lambda \right)=0.35$ and $\sigma_{y}\left(\lambda \right)=0.12$ , respectively. These values were chosen empirically to produce moderate brightness and contrast across sites while limiting saturation of bright pixels from bleached corals or highly reflective substrates. Figure 2 shows examples of raw and color-corrected images from two visits. In addition to the corrected imagery, we also provide the original raw images in the dataset so that users can apply alternative color correction or enhancement methods if desired. The parameters used for the image color correction are provided in Supplementary Materials.

### 2.4 Geometric reconstruction and registration

**FIGURE 3:**
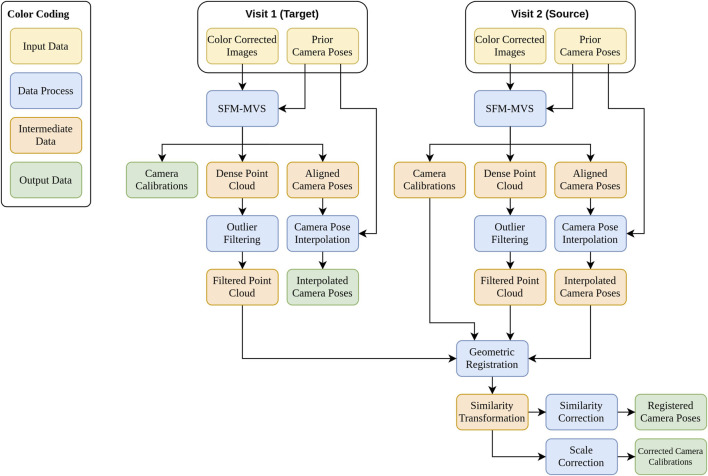
Single line diagram of the geometric reconstruction and registration workflow for an example with two visits. In the example Visit 1 is the registration target, while Visit 2 is the registration source being registered to Visit 1. Note that for the target visit, the SFM-estimated camera calibrations and interpolated camera poses are the final calibrations and camera poses, while for the source visit they are corrected by the similarity transformation estimated by the geometric registration.

To register camera poses across visits to benthic reference sites, we apply a geometric approach based on 3D reconstruction and registration, similar to previous work (Larsen et al., 2023; Boittiaux et al., 2023). Figure 3 outlines the workflow.

We estimate camera calibrations, poses, and scene geometry using structure-from-motion (SFM) and multi-view stereo (MVS). Color-corrected stereo image pairs (Section 2.3) and prior poses from a SLAM system (Mahon et al., 2008) serve as inputs for each visit. SFM and MVS is performed in Agisoft Metashape Professional (Agisoft, 2022), producing globally aligned camera poses, intrinsic and extrinsic calibrations, and dense point clouds. When SFM fails on certain trajectory segments, typically due to poor image overlap or dynamic content, we compute a local similarity transform $S_{s}\in Sim\left(3\right)$ using Umeyama’s least-squares method (Umeyama, 1991). The transform aligns prior poses with neighboring SFM estimates, yielding locally corrected poses. The union of these locally aligned and SFM-aligned poses are referred to as interpolated camera poses.

Once the camera poses for each visit are estimated, we perform geometric registration between the visits to express all camera poses in the same reference frame. For visual localization purposes we require precise relative camera poses across visits, and therefore employ a pairwise geometric registration approach in which we register source visits to a target visit. The geometric registration is done using the dense 3D point clouds derived from MVS. We process the dense point clouds by discarding 3D points observed by fewer than three stereo image pairs, which removes many noisy or weakly constrained points arising from MVS. For each site, we manually select a reference visit (target) as the visit with the greatest spatial overlap with the remaining visits, and all other visits (sources) for that site are registered to this reference. For each source visit, we estimate a 3D similarity transform that we apply to all camera poses of that visit. We also correct the scale of the extrinsic calibration of the stereo rig.

For each site, we define a four-stage registration pipeline. At each stage, we downsample the outlier-filtered point clouds to a prescribed spatial resolution using voxel downsampling and estimate a rigid or similarity transformation between the source and target point clouds. In Stage 1, we perform global registration using fast point feature histogram (FPFH) descriptors (Rusu et al., 2009) and a random sample consensus (RANSAC)-based estimator (Fischler and Bolles, 1981) to obtain an initial similarity transform between the source and target visits. In Stages 2–4, we fix the scale parameter and refine the rotation and translation using colored iterative closest point (ICP) (Park et al., 2017) at progressively finer spatial resolutions, yielding the final similarity transform for that source visit. The final similarity transform is used to update the camera poses and extrinsic calibration for the source visit. This process is repeated for all source visits for a given site. The registration pipeline is implemented using Open3D (Zhou et al., 2018), which provides FPFH, RANSAC-based global registration, and colored ICP routines. The configurations of the registration pipeline are provided in Supplementary Materials. To quantify the registration error between visits, we leverage the Euclidean distance between corresponding points:

$$e_{ij}={\left\|p_{i}-p_{j}\right\|}^{2},\;\left(p_{i},p_{j}\right)\in A,$$ (1)

where $p_{i}$ and $p_{j}$ are corresponding 3D points from the target and source visit, respectively, and A is the set of point cloud correspondences from the final colored ICP stage.

### 2.5 Estimating image seafloor footprints

Estimating image footprints on the seafloor is central to how visual localizations are classified as true or false in this work. Rather than relying on fixed location distance thresholds, which do not account for variations in vehicle altitude and seafloor relief, the proposed approach uses image-footprint overlap to decide whether two images actually observe part of the same seafloor patch. Similar footprint-based reasoning has previously been used in visually augmented navigation (Eustice et al., 2008) and view-based SLAM (Mahon et al., 2008) to propose image-to-image links and loop-closure hypotheses for AUVs. Here, this idea is adapted for long-term VPR by combining calibrated cameras with range estimates to obtain per-view (per-image) 3D footprints. The top-down overlap of these footprints then provides a content-based criterion for defining true localizations.

In this work, we use the following coordinate reference frames:

- The global frame is the WGS84 (i.e., EPSG:4,326) Earth-fixed frame, in which positions are represented by geodetic latitude, longitude, and ellipsoidal height. A 3D point in the global frame is denoted pg .
- The local frame is the 3D Cartesian frame fixed to the environment following North-East-Down (NED) convention. It is defined with respect to a reference point in the global frame, enabling conversion between local coordinates pl and global coordinates pg .
- The vehicle frame is the 3D frame fixed to the vehicle following the SNAME convention (Fossen, 2011), with axes x positive forward, y positive to starboard, and z downward. The origin of the vehicle frame is chosen at the optical center of the left camera of the stereo rig.

Each camera is modeled as an ideal pinhole camera without skew with intrinsics K (Hartley and Zisserman, 2003), and we adopt the OpenCV camera and pixel coordinate conventions (x right, y down, z along the optical axis; pixel coordinates with origin in the top-left corner). A 3D point in the frame of camera view $c_{k}$ at time k is denoted as pck .

#### 2.5.1 Camera model

Under this model, the calibrated intrinsic matrix is

$$K=\left[\begin{array}{ccc} \alpha_{x} & 0 & u_{0}\\ 0 & \alpha_{y} & v_{0}\\ 0 & 0 & 1 \end{array}\right],$$ (2)

where $\alpha_{x}$ and $\alpha_{y}$ are the focal lengths in x and y direction, respectively, and ${\left[u_{0},v_{0}\right]}^{⊤}$ is the principal point of the camera measured in pixels. The projective mapping from a 3D point in the local frame to a 2D point in the pixel frame of camera view $c_{k}$ is given as:

u~j=Kck0p~jck=Kck0Tlckp~jl (3)

Here $u_{j}={\left[u_{j},v_{j}\right]}^{⊤}$ is a point j in the pixel frame of camera view $c_{k}$ , and ${\tilde{u}}_{j}={\left[u_{j}^{⊤},1\right]}^{⊤}$ is its homogeneous representation. $K_{c_{k}}$ is the intrinsic matrix for the camera of view $c_{k}$ and is defined by Equation 2. pjl=xjl,yjl,zjl⊤ is a 3D point expressed in the local frame, and p~jl=pj⊤l,1⊤ is its homogeneous representation. The transformation Tlck=TvcTlvk∈SE3 maps points from the local frame to the frame of camera view $c_{k}$ , combining the vehicle pose Tlvk at time k in the local frame with the time-invariant extrinsics Tvc of the camera c relative to the vehicle. For a calibrated camera, we can invert the projective mapping in Equation 3, and estimate the 3D point on the seafloor corresponding to the image pixel $u_{j}$ with the following equations:

pjck=zj⋅Kck−1u~j (4a)

p~jl=Tcklp~jck (4b)

Here $K_{c_{k}}^{-1}{\tilde{u}}_{j}$ gives the ray direction up to scale in the camera frame, which is then scaled by the range $z_{j}$ . The range is defined as the distance from the optical center $o_{c_{k}}$ of camera view $c_{k}$ to the seafloor along the optical (z-)axis for pixel j . Tckl∈SE3 is the rigid-body transformation from the frame of camera view $c_{k}$ to the local frame.

#### 2.5.2 Range map fusion

Accurate footprint estimation requires spatially consistent range information across the field of view, particularly at image borders where footprint vertices are defined. However, MVS range maps (Section 2.4) are often incomplete, so valid values are frequently missing near image boundaries. In addition, camera views that are unaligned by SFM lack MVS-derived range maps, making them unsuitable for footprint estimation.

To obtain metric range estimates without relying on multi-view overlap, we compute stereo-derived range maps using the calibrated stereo rig and HitNet (Tankovich et al., 2021) for left–right disparity. Stereo reconstruction provides metrically accurate range for individual image pairs and thus supports all camera views, including those not aligned by SFM, but often misses fine structural details and shows geometric inconsistencies where left–right overlap is limited.

To compensate for these shortcomings, we fuse the stereo-derived metric range maps with monocular relative range maps from Depth Anything V2 (Yang et al., 2024), which provide complete but scale-ambiguous predictions. This fusion combines stereo metric accuracy with monocular spatial completeness, yielding dense, metrically consistent range maps for all camera views.

Following recent work on aligning monocular range maps with a global scale and offset (Ganj et al., 2025; Zhang et al., 2025), we adopt a similar strategy to fuse the stereo and monocular estimates. For each image, the stereo range map is first reprojected so that both maps share the same (distorted) pixel frame, and stereo values outside [0.2,6.0] m are masked out to remove close-range artifacts and far-range noise; the same mask is applied to the monocular map. From the remaining pixels, 10% are randomly sampled to form pairs of stereo and monocular ranges, and a global scale and offset $\left(a,b\right)$ are estimated by robust linear regression with a Huber loss:

$$\underset{a,b}{\mathrm{arg min}}\;\underset{i}{\sum }\rho \left({\left\|z_{i}^{fused}-z_{i}^{stereo}\right\|}^{2}\right),$$ (5)

where the fused range for each pixel i is defined as

$$z_{i}^{fused}=a\cdot z_{i}^{rel}+b.$$ (6)

**FIGURE 4:**
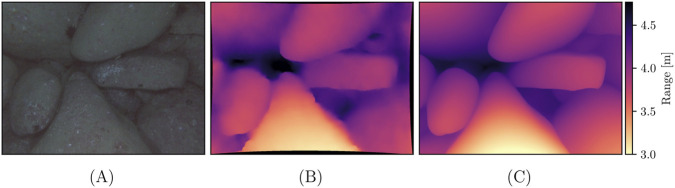
An example showing the fusion of relative and metric stereo-derived range maps for an image from 2009 visit to Site 4. **(A)** The color corrected image, **(B)** the stereo range map reprojected into the pixel coordinate system of the original image, and **(C)** the range map estimated by fusing the relative range map with the stereo-derived range map. The stereo-derived and fused range maps are rendered with the same color map to indicate the range. Overall, the fused range maps show sharper edges and do not exhibit the geometric inconsistencies on the left side seen in the stereo range map, which are believed to be caused by limited left–right image overlap.

The parameters $\left(a,b\right)$ obtained by solving Equation 5 are applied to the full-resolution monocular range map using Equation 6, yielding a dense, metrically scaled range map. This range map is then used for footprint estimation. Figure 4 shows an example with the stereo range map and fused range map for an image captured by the left (RGB) camera of the stereo rig.

#### 2.5.3 Image footprint and overlap estimation

In our approach, we estimate the image footprints by using the inverse projective mapping in Equation 4a for the corners of each image. We estimate the range $z_{j}$ for each corner j as the median range in a 30×30 pixel patch in the corresponding corner of the fused range maps from Section 2.5.2. Using Equation 4a with the pixel coordinates $u_{j}$ and range estimate $z_{j}$ for each image corner, and transforming the 3D points using Equation 4b, we obtain the set of 3D points (3D polygon) in the local frame Pckl representing the image footprint for camera view $c_{k}$ :

Pckl=Pck,1l,Pck,2l,Pck,3l,Pck,4l (7)

To perform spatial operations on the image footprints, we georeference them by converting the footprints expressed in the local NED frame, Pckl , to footprints expressed in global WGS84 frame, Pckg . This is primarily a choice of convenience as we no longer need to keep track of the reference point for the local frame. Coordinate conversion between the local NED frame and the global frame are done with PyMap3D version 3.1.0 (Hirsch, 2018), which uses a local tangent plane approximation. A table with the origins of the local NED reference frames can be found in Supplementary Materials. Subsequently, all spatial operations are performed on the footprints in the global frame.

To perform spatial operations between image footprints, the 3D footprints are projected to 2D by discarding the ellipsoidal height. This approximation is valid for the near-horizontal seafloor sites in SEALOC, but breaks down in environments where the surfaces of interest are primarily vertical, such as underwater cliffs or caves. Let P¯g denote the 2D conversion of the 3D footprint Pg in the global frame. For the 2D footprints P¯ckg and P¯cjg of camera view $c_{k}$ and $c_{j}$ , respectively, we denote the spatial intersection and spatial union operators as:

P¯ckg∩sP¯cjg=SpatialIntersectP¯ckg,P¯cjg (8a)

P¯ckg∪sP¯cjg=SpatialUnionP¯ckg,P¯cjg (8b)

For non-overlapping footprints $SpatialIntersect\left(\cdot \right)$ returns an empty set. To detect if camera view $c_{k}$ and $c_{j}$ have overlapping footprints, we check that the spatial intersection between their 2D footprints, P¯ckg and P¯cjg , respectively, is not an empty set, i.e.,:

P¯ckg∩sP¯cjg≠∅ (9)

In order to quantify the amount of overlap of image footprints relative to the size of the footprints, we define the footprint intersection over union (IoU) as:

IoUkj=AreaP¯ckg∩sP¯cjgAreaP¯ckg∪sP¯cjg (10)

**FIGURE 5:**
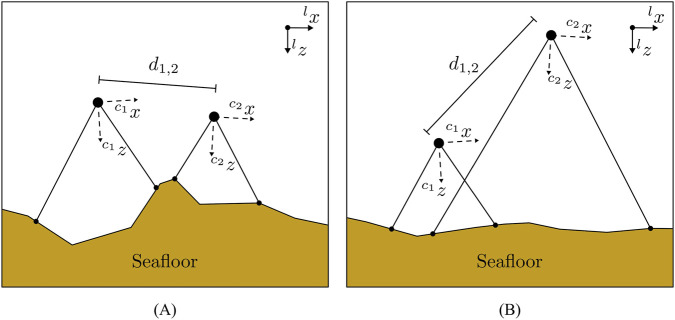
Illustration of a 2D simplified model of our footprint estimation method for two scenarios. Panel **(A)** shows a scenario where local terrain relief causes non-overlapping image footprints for two spatially close camera views. Panel **(B)** shows a scenario where large altitude differences causes spatially distant camera views to have overlapping image footprints.

Here $Area\left(\cdot \right)$ is the function that returns the spatial area of a 2D polygon. The spatial intersect and union operators in Equations 8a, 8b, and the Area(·) function, are implemented with Shapely (Gillies and contributors, 2023) which uses GEOS (GEOS contributors, 2025) in its backend to perform geometric operations. Figure 5 shows a simplified 2D model of our footprint estimation method for two scenarios. Panel (A) shows a scenario where local terrain relief causes non-overlapping image footprints for two spatially close camera views. Panel (B) shows a scenario where large altitude differences cause spatially distant camera views to have overlapping image footprints.

### 2.6 Evaluating long-term visual place recognition

In this paper, we treat long-term VPR as an image retrieval problem: each image is mapped into a vector representation $e\in R^{d}$ , known as an image descriptor or embedding, and image-level nearest-neighbor search in descriptor space is used to retrieve candidate images from a database of images with associated location estimates.

#### 2.6.1 Selected visual place recognition models

To benchmark long-term VPR on the SEALOC dataset, we evaluate eight representative global-descriptor models selected to cover a broad spectrum of modern VPR approaches, spanning both convolutional neural network (CNN)- and vision transformer (ViT)-based architectures. An overview of all models, including their backbone and key methodological contribution, is provided in Supplementary Materials.

The CNN-based methods represent a progression of global descriptor designs built on convolutional backbones. NetVLAD (Arandjelovic et al., 2016) introduced a VLAD aggregation layer for end-to-end training, while CosPlace (Berton et al., 2022) and EigenPlaces (Berton et al., 2023) improved training scalability and viewpoint robustness through classification-based objectives. More recently, MixVPR (Ali-bey et al., 2023) proposed a lightweight MLP aggregator, achieving competitive performance with reduced computational overhead.

The ViT-based methods all leverage DINOv2 (Oquab et al., 2024) features but differ in aggregation and training strategy. AnyLoc (Keetha et al., 2024) applies unsupervised VLAD aggregation on frozen features for zero-shot VPR. SALAD (Izquierdo and Civera, 2024) and CliqueMining (Izquierdo and Civera, 2025) fine-tune the backbone using optimal transport-based aggregation, with CliqueMining further improving short-range sensitivity via graph-based batch sampling. MegaLoc (Berton and Masone, 2025) extends this with large-scale multi-dataset training and achieves SOTA performance across multiple retrieval tasks.

#### 2.6.2 Evaluation protocol and metrics

All VPR models are evaluated on color-corrected RGB images from the left camera of the stereo rig (Section 2.3), matching the single-view RGB input configuration for which all compared methods were designed and benchmarked. Since the sites in the SEALOC dataset exhibit different seafloor characteristics, we evaluate the VPR models on a per-site basis. For each site, we split the visits into pairs $\left(V_{Q},V_{D}\right)$ , where $V_{Q}$ represents a new unseen visit and $V_{D}$ represents a previously seen visit. We refer to $V_{Q}$ and $V_{D}$ as the query and database visit to reflect their role when performing image retrieval. We use a temporal pairing strategy for the visits, where a query visit is only paired with visits that were conducted before it in time.

For each visit pair, we denote the set of camera views from the query and database visit as $C_{Q}$ and $C_{D}$ , respectively. Here we introduce the concept of a link, which is a ground-truth correspondence between two camera views. The set of camera view links composes our ground truth, i.e., what we consider to be true localizations. For every camera view in the query visit, we create links to the camera views in the database visit by using the georeferenced 3D image footprints estimated with the methodology in Section 2.5.3. We use the criterion in Equation 9 to find pairs of query and database camera views with overlapping image footprints. For query and database camera views with overlapping footprints, we create a set of camera view links, denoted $L_{Q,D}$ by setting a lower threshold $\tau_{f}$ for the footprint IoU defined in Equation 10. The set of camera view links is defined as:

$$L_{Q,D}=\left\{\left(c_{q},c_{d}\right)|{IoU}_{qd}>\tau_{f},\;c_{q}\in C_{Q},\;c_{d}\in C_{D}\right\}$$ (11)

To design a conservative lower footprint IoU threshold $\tau_{f}$ that makes us confident that two camera views have overlapping footprints, we consider a simplified scenario where both cameras observe a flat seafloor patch from the same altitude while looking straight down, with their footprints aligned along the largest side. Under the assumption that the registration error is a purely horizontal translation, normal to the aligned footprint side and smaller than the smallest footprint side $L_{f}$ , the induced footprint IoU is

$${IoU}_{e}=\frac{W_{f}\cdot t_{e}}{2\cdot W_{f}\cdot L_{f}-W_{f}\cdot t_{e}}=\frac{t_{e}}{4\cdot a\cdot \tan\left(0.5\cdot FOV\right)-t_{e}},$$ (12)

where $t_{e}$ is the horizontal translation and a is the altitude. $W_{f}$ and $L_{f}$ are the lengths of the largest and smallest footprint sides, respectively. FOV is the field of view along the axis corresponding to $L_{f}$ . With this simplified model, choosing $\tau_{f}={IoU}_{e}$ makes us confident that the two footprints overlap for registration errors up to $t_{e}$ . The specific threshold value derived for the SEALOC dataset is presented in Section 3.2.

For each of the RGB images associated with the camera views we use the VPR model to create the set of image descriptors $E_{Q}$ and $E_{D}$ for the query and database camera views, respectively. For every query camera view $c_{q}$ , we perform a similarity search between the image descriptor of the query camera view, $e_{q}$ , and the set of image descriptors for the database camera views, $E_{D}$ . The similarity search finds the K candidates from the database image descriptors that minimize the L2 distance to the image descriptor of the query camera view $e_{q}$ . The similarity search is defined as:

$$C_{q,p}^{\left(K\right)}=\left\{c_{p}^{\left(1\right)},\ldots ,c_{p}^{\left(K\right)}\right\}=\underset{c_{p}\in C_{D}}{TopK}\left(-{\left\|e_{q}-e_{p}\right\|}^{2}\right)$$ (13)

Here $C_{q,p}^{\left(K\right)}$ is the ordered set of candidate database camera views for query camera view $c_{q}\in C_{Q}$ , $e_{q}\in E_{Q}$ is the image descriptor of the query camera view, and $e_{p}\in E_{D}$ is the image descriptor of the candidate database camera view $c_{p}\in C_{D}$ . The k -th nearest candidate database camera view is denoted $c_{p}^{\left(k\right)}$ . We refer to k as the retrieval or candidate rank, and K as the rank cutoff. We implement $TopK\left(\cdot \right)$ using FAISS (Douze et al., 2025).

For each visit pair, we evaluate localizations for each query camera view using the set of retrieved database camera views, $C_{q,p}^{\left(K\right)}$ , defined in Equation 13, and the set of footprint-based ground-truth links between the query and database camera views, $L_{Q,D}$ , defined in Equation 11. We consider a VPR candidate as a true positive if the pair of query and database camera views $\left(c_{q},c_{p}\right)\in C_{q,p}^{\left(K\right)}$ matches the ground-truth links, i.e., $\left(c_{q},c_{p}\right)\in L_{Q,D}$ . Consequently, a pair of query and database camera view in the ground-truth links is considered a false negative if it is not in the VPR retrieved set, i.e., $\left(c_{q},c_{d}\right)\notin C_{q,p}^{\left(K\right)}$ .

We adopt the VPR-specific definition of Recall@K to ensure comparability with standard VPR benchmarks, where this metric is the prevailing evaluation metric. Recall@K directly quantifies the proportion of query images that have at least one correct match within the top-K retrieved candidates, which provides an intuitive measure of localization success and corresponds to what is typically referred to as HitRate@K in information retrieval. Formally, we define Recall@K as

$$Recall@K=\frac{\text{Number of queries with at least one correct candidate}}{\text{Number of valid queries}}$$ (14)

Here, a query is considered recognized if any of the retrieved pairs $\left(c_{q},c_{p}\right)\in C_{q,p}^{\left(K\right)}$ is a true positive, i.e., $\left(c_{q},c_{p}\right)\in L_{Q,D}$ . We consider unlinked query camera views, i.e., query camera views that do not have any database camera views with overlapping footprints, as invalid queries. Consequently, we do not consider the VPR retrievals for those queries when calculating Recall@K .

While Recall@K evaluates recognition success on a per-query basis, IRRecall@K follows the classical formulation from information retrieval, measuring the proportion of all relevant query–database pairs that are correctly retrieved. We use IRRecall@K specifically when studying how different definitions of ground truth affect retrieval performance. The information retrieval recall IRRecall@K is defined as

$$IRRecall@K=\frac{TP@K}{TP@K+FN@K},$$ (15)

where TP@K and FN@K denote, respectively, the number of true-positive and false-negative query–database pairs within the top-K retrieved candidates.

## 3 Results

This section evaluates the proposed dataset construction and long-term visual localization framework across the five benthic reference sites. We first validate the multi-visit geometric registration and the footprint-based ground truth used to relate camera views. We then characterize the footprint-based linking of camera views, and analyze how its parameters affect the resulting link statistics. Next, we assess the performance of state-of-the-art VPR models across habitat types and revisit intervals, including within-site spatial patterns. Finally, we compare footprint-based and location-based ground-truthing strategies and examine how they influence the reported VPR performance metrics.

### 3.1 Geometric registration

**FIGURE 6:**
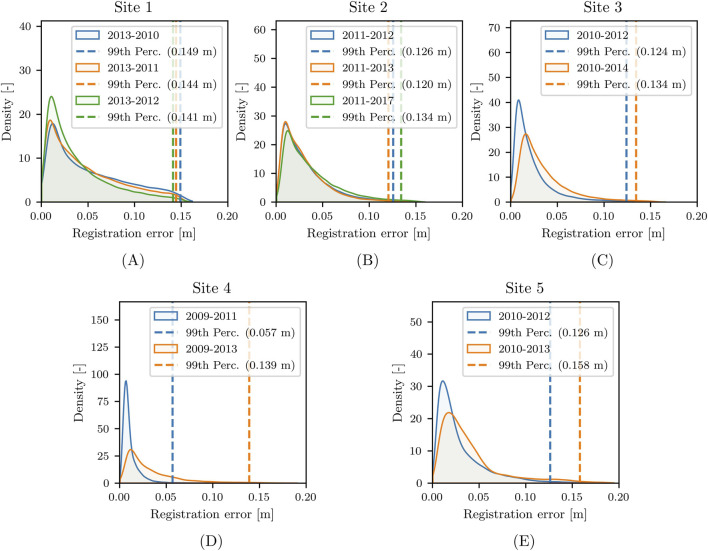
Distribution of geometric registration errors for the visit pairs at **(A)** Site 1, **(B)** Site 2, **(C)** Site 3, **(D)** Site 4, and **(E)** Site 5. The legends are on the format “Target-Source” and indicate the year of the target and source visit for each visit pair used in the geometric registration.

In this section, we report the geometric registration errors for the benthic reference sites, computed according to Equation 1, for the point clouds and point correspondences from with the reconstruction and registration pipeline described in Section 2.4. Figure 6 shows the distribution of registration errors for all target–source visit pairs at each site. Across sites, the bulk of the errors are on the order of a few centimeters, and the 99th percentile error remains below 0.16 m for all visit pairs, indicating that the visits are consistently aligned at sub-decimeter accuracy. The error distributions differ between sites: Site 1 and Site 2 exhibit heavier tails towards larger errors, whereas Sites 3–5 show more concentrated distributions with fewer large-error outliers.

### 3.2 Linking camera views with overlapping image footprints

To estimate a conservative lower footprint IoU threshold, $\tau_{f}$ , for which we are confident that two camera views overlap despite registration errors, we use Equation 12. We obtain the camera field of view along the y-axis from the calibration procedure in Section 2.4, giving ${FOV}_{y}=34$ deg. For the camera altitude, we set a=2.0 meters, which is approximately the lowest average altitude across visits in the SEALOC dataset. For the registration error, we set $t_{e}=0.16$ meters, which exceeds the 99th-percentile registration error for all visits. With these parameter choices, Equation 12 yields a lower footprint IoU threshold of $\tau_{f}\approx 0.07$ , which we adopt for the remainder of the paper.

Table 2 shows the visit pairs along with various geometry statistics across the benthic reference sites. The visit pairs have been created using the temporal pairing strategy outlined in Section 2.6.2, and the footprint-based links have been created with the methodology in Section 2.5. The footprint-based links between query and database camera views have been filtered with the lower footprint IoU threshold $\left(\tau_{f}=0.07\right)$ .

**TABLE 2:** Overview of the visit pairs and footprint links filtered with $\tau_{f}=0.07$ . The visit pairs are created using the temporal pairing strategy outlined in Section 2.6.2.

Site	Visit years	Database view count	Query view count	Avg.database view Alt	Avg.query view Alt	Query coverage overlap	Link count	ALQ*	Avg.footprint width	Loc.dist. 95th perc
-	-	-	-	m	m	-	-	-	m	m
Site 1	2010–2011	2,323	2,255	2.04	2.04	74%	27.8 K	15	1.65	1.37
	2010–2012	2,323	2,184	2.04	2.00	53%	14.8 K	13	1.65	1.37
	2010–2013	2,323	2,134	2.04	1.99	65%	21.1 K	15	1.64	1.37
	2011–2012	2,255	2,184	2.04	2.00	65%	16.4 K	10	1.64	1.41
	2011–2013	2,255	2,134	2.04	1.99	80%	21.0 K	11	1.63	1.36
	2012–2013	2,184	2,134	2.00	1.99	75%	18.1 K	10	1.62	1.33
Site 2	2011–2012	2,317	2,225	2.27	2.10	80%	23.9 K	14	1.82	1.99
	2011–2013	2,317	2,164	2.27	2.00	87%	24.7 K	13	1.79	2.04
	2011–2017	2,317	3,467	2.27	2.42	82%	50.4 K	16	1.95	2.32
	2012–2013	2,225	2,164	2.10	2.00	82%	23.9 K	11	1.69	1.65
	2012–2017	2,225	3,467	2.10	2.42	72%	34.1 K	12	1.86	2.18
	2013–2017	2,164	3,467	2.00	2.42	75%	31.4 K	11	1.83	2.25
Site 3	2010–2012	2,728	2,597	2.14	2.01	66%	24.4 K	12	1.64	1.47
	2010–2014	2,728	2,246	2.14	2.24	61%	20.0 K	13	1.71	1.54
	2012–2014	2,597	2,246	2.01	2.24	66%	18.7 K	11	1.67	1.56
Site 4	2009–2011	6,280	2,267	3.24	2.48	96%	77.8 K	35	2.26	2.48
	2009–2013	6,280	2,134	3.24	2.26	79%	60.8 K	31	2.07	2.46
	2011–2013	2,267	2,134	2.48	2.26	57%	19.4 K	15	1.82	1.97
Site 5	2010–2012	2,701	3,687	2.20	2.13	76%	50.5 K	16	1.82	1.87
	2010–2013	2,701	3,313	2.20	2.32	82%	49.0 K	17	1.87	1.85
	2012–2013	3,687	3,313	2.13	2.32	87%	73.2 K	24	1.88	1.95

* Average link per valid query.

The query coverage overlap, defined as the fraction of query coverage area overlapped by the database visit (area intersection divided by query coverage area), ranges from approximately 50%–95%. The average camera altitude varies between approximately 2.0 and 3.2 m across visit pairs and sites. The visits to Site 4 have the largest average altitude difference (∼0.8 m between the 2009 and 2011 visits) and the highest absolute average altitude (∼3.2 m for the 2013 visit).

The average number of footprint-based links per query camera view varies substantially across visit pairs, from about 10 to 35 links per query, reflecting differences between sites and visit pairs in terms of terrain ruggedness, survey layout, and camera altitudes. Consequently, the 95th percentile location distance between the linked camera views varies, with values ranging between 1.3 and 2.5 m.

### 3.3 Evaluating long-term visual place recognition performance

In this subsection, we evaluate long-term VPR performance across all reference sites and visit pairs in the SEALOC dataset using our footprint-based ground truth. We first compare how SOTA VPR models perform across different seafloor types (Section 3.3.1) and analyze within-site spatial patterns for the localization results of a selected model (Section 3.3.2). Throughout Section 3.3, VPR candidates are retrieved for every query using Equation 13, and Recall@K is calculated using Equation 14.

#### 3.3.1 Evaluating visual place recognition performance across sites

**FIGURE 7:**
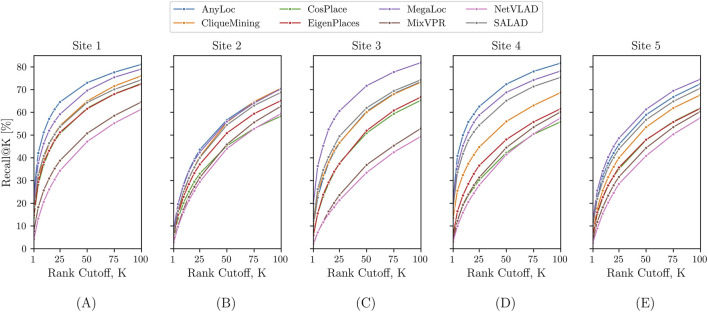
Mean Recall@K across all visit pairs at **(A)** Site 1, **(B)** Site 2, **(C)** Site 3, **(D)** Site 4, and **(E)** Site 5. Results are based on the footprint-based ground truth from Section 3.2.

To assess how the selected VPR models generalize across seafloor types, we evaluate their mean Recall@K across all visit pairs for each reference site. This provides insight into how seafloor characteristics influence localization performance. As shown in Figure 7, we report the mean Recall@K over a range of rank cutoffs K , illustrating how retrieval accuracy varies with the number of candidates considered. Ground-truth correspondences are defined using footprint-based camera links established through the methodology in Section 2.6.2, applying the lower footprint IoU threshold $\left(\tau_{f}=0.07\right)$ derived in Section 3.2 to compensate for residual registration errors between visits.

The mean recall curves show significant variation in VPR performance across sites. Site 1, Site 3, and Site 4 exhibit higher mean recall across the entire range of K compared to the other sites. Recall is moderately lower at Site 5, although the separation between models remains clear, whereas Site 2 yields the lowest recall with only minor differences between models. The relative differences in recall between higher- and lower-performing models are most clear at lower rank cutoffs K . AnyLoc and MegaLoc are generally the two strongest models and consistently achieve the highest recall across most sites. MegaLoc is particularly strong at Site 3, where it achieves considerably higher recall than all other models. We also observe that the ViT-based models (AnyLoc, CliqueMining, MegaLoc, and SALAD) consistently achieve higher recall than the CNN-based models (CosPlace, EigenPlaces, MixVPR, and NetVLAD) across all sites.

For practical VPR applications, performance at lower rank cutoffs is particularly relevant, since the system typically only considers a small set of top-ranked candidates for downstream verification. Consequently, we focus on two representative operating points and report Recall@1 and Recall@10 for each visit pair, as well as the mean Recall@1 and Recall@10 across all visit pairs to each site in Table 3. This complements the trends observed in Figure 7 by quantifying how often each model retrieves the correct place as the top match or within the top 10 candidates.

**TABLE 3:** Recall@1 and Recall@10 for all VPR models and visit pairs in the dataset, as well as mean across all visit pairs to each site. For each rank cutoff K and visit pair, we use green and red to highlight the best and second best model, respectively. Results are based on the footprint-based ground truth from Section 3.2.

Site	Visit years	AnyLoc		CliqueMining		CosPlace		EigenPlaces		MegaLoc		MixVPR		NetVLAD		SALAD	
		R@1	R@10	R@1	R@10	R@1	R@10	R@1	R@10	R@1	R@10	R@1	R@10	R@1	R@10	R@1	R@10
Site 1	2010–2011	42.3	73.0	30.3	56.2	20.1	49.1	21.0	49.3	31.3	59.5	12.6	35.7	5.3	29.1	30.4	54.8
	2010–2012	20.9	50.9	14.4	40.0	14.0	36.2	13.2	37.4	19.2	47.2	6.2	22.1	2.9	18.3	15.2	41.2
	2010–2013	15.2	42.3	10.8	34.1	10.3	33.0	12.4	35.6	13.1	36.3	7.4	28.8	4.5	20.8	13.7	37.2
	2011–2012	24.2	47.6	16.7	39.0	11.6	32.9	12.6	33.4	23.0	46.1	6.6	21.3	3.6	16.8	19.4	40.8
	2011–2013	14.2	35.7	8.3	27.6	10.2	27.7	10.9	29.2	13.8	33.9	4.7	19.7	4.8	18.5	10.0	28.8
	2012–2013	32.5	57.7	21.9	42.1	16.7	43.5	18.2	43.2	31.1	54.7	9.1	26.3	4.0	20.3	22.4	42.1
	Mean	24.9	51.2	17.0	39.8	13.8	37.1	14.7	38.0	21.9	46.3	7.8	25.6	4.2	20.7	18.5	40.8
Site 2	2011–2012	9.5	32.7	5.9	28.0	4.8	23.8	6.9	30.6	11.1	32.4	2.2	16.7	2.9	16.9	6.9	29.9
	2011–2013	7.2	28.7	5.8	23.1	3.4	16.4	3.5	20.1	8.2	27.6	2.7	15.5	2.3	14.7	5.5	23.9
	2011–2017	5.5	26.1	3.6	21.0	3.3	17.5	3.7	18.1	5.8	22.7	1.9	17.4	3.3	14.4	3.8	20.9
	2012–2013	7.7	27.8	8.0	26.1	4.6	18.9	5.6	22.7	10.3	28.9	3.3	18.4	2.8	16.7	8.0	27.8
	2012–2017	6.8	24.8	4.6	23.1	3.7	19.1	4.6	22.8	7.0	23.9	3.1	19.1	2.2	13.9	6.1	24.0
	2013–2017	7.7	29.1	5.9	26.0	6.3	24.6	5.0	22.9	9.8	30.8	2.4	16.4	3.3	20.0	6.6	24.4
	Mean	7.4	28.2	5.6	24.6	4.3	20.1	4.9	22.8	8.7	27.7	2.6	17.3	2.8	16.1	6.1	25.1
Site 3	2010–2012	16.8	42.5	13.2	35.8	7.2	26.5	7.9	27.5	23.7	49.3	3.3	13.4	2.5	12.3	13.5	36.8
	2010–2014	6.7	25.6	8.4	26.0	3.6	19.0	3.7	20.1	13.6	37.2	1.4	10.4	1.7	9.3	9.1	31.9
	2012–2014	6.5	24.4	11.1	34.9	5.7	23.2	6.2	23.7	22.1	49.4	1.4	12.2	2.5	13.4	13.8	35.2
	Mean	10.0	30.8	10.9	32.3	5.5	22.9	6.0	22.7	19.8	45.3	2.0	12.0	2.2	11.7	12.2	34.7
Site 4	2009–2011	24.8	57.6	14.2	33.9	5.3	19.9	6.2	22.2	27.3	56.2	5.9	25.4	4.1	21.2	21.9	48.1
	2009–2013	21.0	48.1	17.0	38.8	6.0	21.1	6.6	25.4	23.3	47.9	3.4	15.7	3.1	14.9	22.8	49.9
	2011–2013	18.4	44.2	9.4	24.6	3.5	16.2	5.2	22.8	12.1	31.9	2.5	16.8	2.3	11.7	9.5	27.3
	Mean	21.4	49.9	13.5	32.4	4.9	19.1	6.0	23.5	20.9	45.3	4.0	19.3	3.1	15.9	18.1	41.8
Site 5	2010–2012	7.8	27.7	4.7	19.9	6.0	23.9	4.9	20.5	9.4	28.6	3.6	17.2	2.2	13.0	7.3	24.4
	2010–2013	8.5	28.3	6.5	24.5	4.7	18.5	5.4	19.7	10.5	31.9	3.6	17.0	2.3	14.4	10.1	30.2
	2012–2013	13.9	39.6	10.4	32.4	6.2	24.6	7.6	27.9	17.9	42.2	4.5	20.5	3.4	19.0	13.2	34.2
	Mean	10.1	31.9	7.2	25.6	5.7	22.3	5.9	23.8	12.6	34.2	3.9	18.2	2.6	15.5	10.2	29.6

Overall, the numbers in Table 3 confirm the trends observed in Figure 7, with AnyLoc and MegaLoc achieving the highest Recall@1 and Recall@10 on most sites. In particular, AnyLoc performs slightly better than MegaLoc on Site 1, achieving mean Recall@1 and Recall@10 of 24.9% and 51.2%, respectively, compared to 21.9% and 46.3% for MegaLoc. Conversely, MegaLoc is considerably stronger on Site 3, with mean Recall@1 and Recall@10 of 19.8% and 45.3%, whereas AnyLoc achieves 10.0% and 30.8%, respectively.

#### 3.3.2 Within-site spatial visual place recognition patterns

In order to highlight spatial patterns in VPR performance attributable to seafloor and motion characteristics, we present maps with the localization results for MegaLoc, which is the best overall performing model according to Section 3.3.1. We show localization results using a limited number of VPR candidates per query image, with a rank cutoff of K=5 . This reflects a setting where only a small number of top-ranked candidates are considered for downstream verification.

**FIGURE 8:**
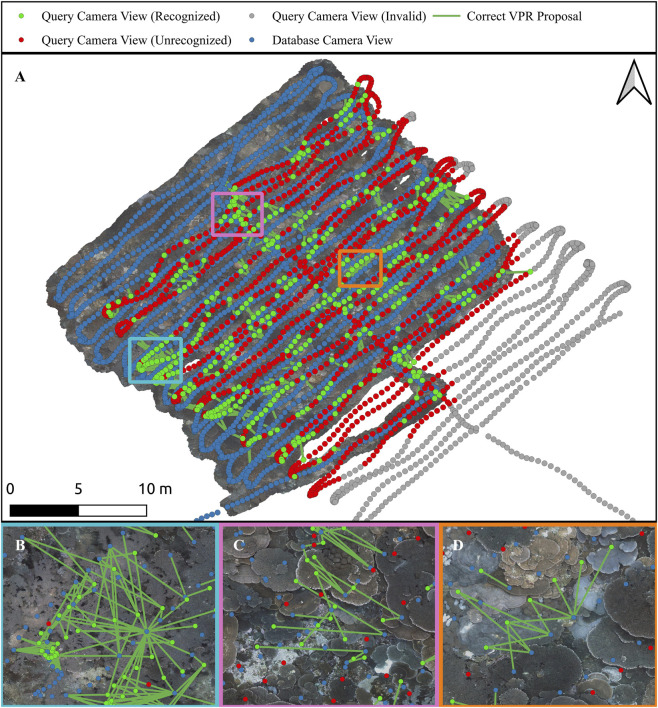
Visual place recognition results at Site 1 rendered on top of an orthomosaic derived from the 2010 images, using MegaLoc with K=5 to retrieve 2010 database images for 2013 query images. **(A)** Overview of place recognition results for the entire site. **(B)** Cluster of recognized query camera views at the end of a survey leg. **(C)** Cluster of recognized query camera views at a cross-over point between survey legs. **(D)** Trajectory segment with multiple consecutively recognized query camera views.

**FIGURE 9:**
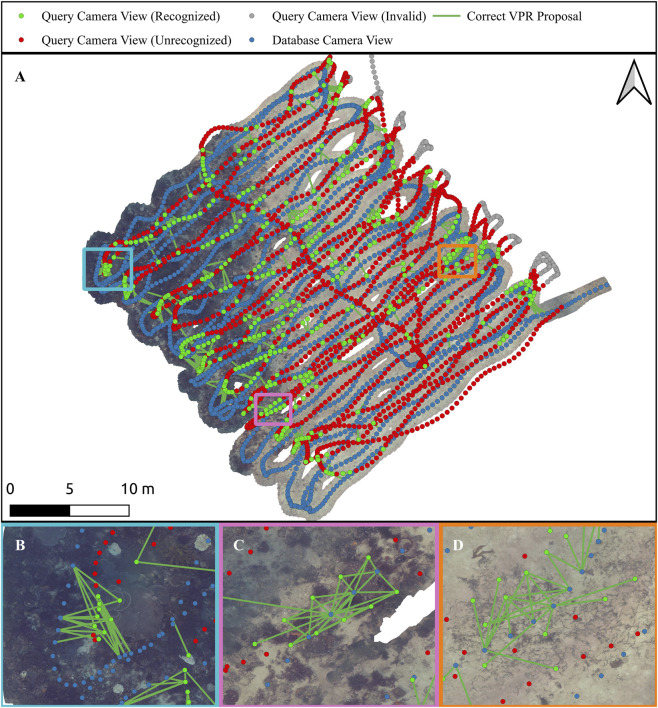
Visual place recognition results at Site 2 rendered on top of an orthomosaic derived from the 2011 images, using MegaLoc with K=5 to retrieve 2011 database images for 2013 query images. Panel **(A)** show an overview of place recognition results for the entire site. Panel **(B)** shows a cluster of recognized query camera views at the end of a survey leg. Panel **(C)** shows trajectory segments with recognized query camera views as the AUV passes over coral colonies in the transition between the soft sediment bottom and the dense coral reef. Panel **(D)** shows trajectory segments with recognized query camera views as the AUV passes over a seagrass meadow on the soft sediment bottom.

Figure 8 shows a map with the localization results for the 2010–2013 visit pair to Site 1 using MegaLoc. Each query camera view is labeled as recognized (at least one correct proposal in the top K ), unrecognized (no correct proposals), or invalid (no ground-truth match), according to the protocol in Section 2.6.2. Correct VPR proposals are visualized as links between recognized query views and their matched database views. Figure 9 shows the corresponding localization results for the 2011–2013 visit pair to Site 2. Similar maps with localization results for visit pairs to Site 3, 4, and 5 can be found in Supplementary Materials.

In Figure 8A, recognized queries are distributed throughout Site 1 but typically appear in spatial clusters of 2–5 consecutive views along the trajectory. Panel (B) shows a zoom-in on the end of a survey leg where the vehicle turns, with a high concentration of recognized queries. In (C), recognized queries cluster at a cross-over point between survey legs. In (D), multiple consecutive recognized queries are matched to a parallel trajectory segment.

Figure 9A provides an overview of the localization results throughout Site 2. The western part of the site covers a dense coral reef, the eastern part consists of soft sediment bottom, and a transition zone with sparse corals lies between them. The overview reveals a clear difference in localization performance between these regions, where recognized queries are much more frequent over areas with corals, whereas they are less common over the soft sediment bottom. Panel (B) shows large concentrations of recognized queries at a survey leg end, similar to Site 1. In (C), multiple trajectory segments with recognized queries appear over seafloor patches with sparse corals in the transition zone. In (D), trajectory segments with recognized queries lie over a seagrass meadow on the soft sediment bottom.

Across both Figures 8, 9, recognized queries are typically spatially clustered, either around patches with distinctive seafloor features or along trajectory segments where the motion produces many images with overlapping footprints. Figures showing recognized query–database image pairs for the visit pairs in Figures 8, 9 are provided in Supplementary Materials. These figures illustrate distinctive seafloor features associated with successful VPR proposals and highlight scene changes between the database and query visits.

#### 3.3.3 Evaluating visual place recognition performance across revisits

**FIGURE 10:**
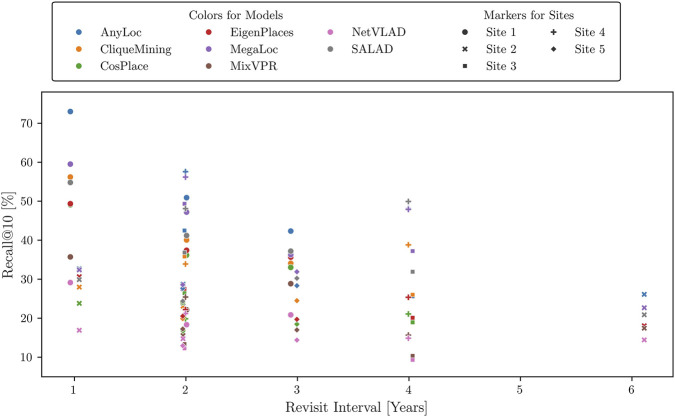
Recall@10 for all VPR models for varying revisit intervals to the sites in the dataset. The revisit intervals are in absolute time differences. The overall trend shows a decline of VPR Recall@10 with increasing revisit intervals. For Site 1 and Site 2, Recall@10 shows a sharp decline between revisit intervals of 1 and 2 years, before a more gradual decline. For Site 5, Recall@10 increases between revisit intervals of 2 and 3 years, indicating that additional factors affect Recall@10. Results are based on the footprint-based ground truth from Section 3.2.

To evaluate how VPR performance is affected by increasing revisit interval, i.e., the time between two visits, we report Recall@10 for visit pairs where the first visit to each site serves as the database visit. In Figure 10, the general trend is that Recall@10 decreases with increasing revisit interval. For the sites with lower overall recall, Site 2 and Site 5, this trend is less pronounced, and Recall@10 remains at similar levels across a range of revisit intervals. For sites with more than three visits, i.e., Site 1 and Site 2, we observe a sharper decline between revisit intervals of 1 and 2 years, followed by a more gradual decline towards a plateau at longer intervals.

### 3.4 Evaluating differences in ground-truthing strategies

To quantify how footprint-based and location-based ground-truthing strategies affect VPR performance metrics, we analyze the performance of the VPR models under two ground-truth definitions, one footprint-based and one location-based. For the footprint-based ground truth, we use the lower footprint IoU threshold $\left(\tau_{f}=0.07\right)$ as established in Section 3.2. For each visit pair, we set the distance threshold for the location-based ground truth to the 95th percentile of distances between footprint-linked camera views. This approach makes the location-based ground truth for each visit pair largely overlap with the footprint-based one, while excluding the most extreme spatial outliers. However, because the location-based ground truth does not explicitly enforce footprint overlap or camera field of view constraints, it also introduces additional links that the footprint-based method deliberately excludes (see Panel (A) in Figure 5).

As in Section 3.3, we retrieve VPR candidates for every query using Equation 13. To compare the VPR models for the two ground truths, we use the VPR-specific definition of recall, Recall@K , defined in Equation 14, and additionally the information-retrieval definition of recall, IRRecall@K , defined in Equation 15. We use both recall definitions because VPR recall is not penalized for false negatives since adding more ground-truth links can only increase the probability that a query is counted as recognized. In contrast, information-retrieval recall IRRecall@K is penalized for false negatives, and therefore decreases when additional relevant query–database pairs are not retrieved. The VPR recall Recall@K and information-retrieval recall IRRecall@K for K=10 are denoted R@10 and IR@10, respectively.

**FIGURE 11:**
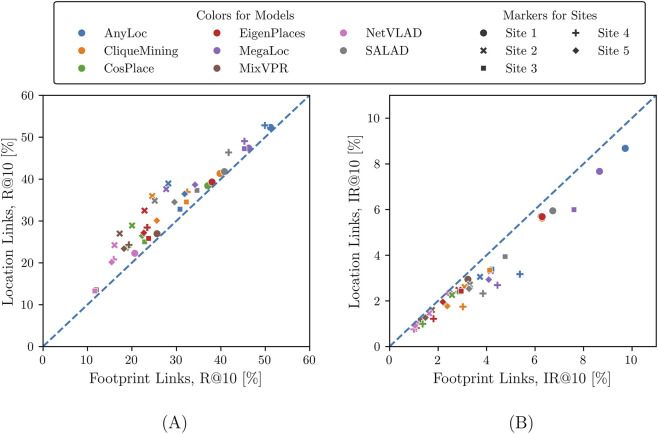
Scatter plots of **(A)** R@10 and **(B)** IR@10 for the footprint-based and location-based ground truth. The R@10 is consistently higher for the location-based ground truth for all sites and VPR models, and indicates that model performance is over-estimated with the location-based ground truth. IR@10 is consistently higher for the footprint-based ground truth for all sites and models, and indicate that the VPR retrievals are more aligned with the footprint-based links.

Figure 11 shows R@10 (A) and IR@10 (B) across all VPR models for the footprint-based and location-based ground truths, averaged over site visit pairs. R@10 is consistently higher under the location-based ground truth, whereas IR@10 is consistently higher under the footprint-based ground truth, reflecting the different ways the two recall definitions respond to additional ground-truth links. Examining the R@10 scatter plot, points for each site cluster along lines with a roughly constant offset from the diagonal. This pattern indicates that the R@10 offsets between the two ground truths are largely specific to each site and relatively consistent across both strong and weak VPR models. For IR@10, the points do not follow a roughly constant offset from the diagonal. Instead, stronger models exhibit a larger deviation from the diagonal than weaker models, indicating that they retrieve a larger portion of the footprint-based links than of the additional links introduced by the location-based ground truth. A detailed plot of R@10 and IR@10 as functions of the distance threshold for the location-based ground truth is provided in Supplementary Materials.

To relate the differences in performance metrics to the geometry of the two ground truths, we report the metrics for MegaLoc and NetVLAD, which are considered the strongest and weakest model, for each visit pair together with key geometric statistics for the footprint-based and location-based ground truth. Table 4 shows the average footprint widths and distance thresholds, and the average link per query (ALQ) for the two ground truths. Note that a link means a ground-truth correspondence in this context. Across sites, the location-based ground truth yields a higher ALQ than the footprint-based ground truth. At Site 1, the distance threshold is close to the effective footprint width and ALQ increases only slightly, whereas at Sites 2, 4, and 5 the threshold substantially exceeds the footprint width and location-based ALQ increases markedly. The overall higher ALQ for the location-based ground truth indicates that it includes numerous query–database links that are not present under the more conservative footprint-based ground truth.

**TABLE 4:** Summary of geometric statistics and retrieval performance for MegaLoc and NetVLAD under footprint-based and location-based ground truth. Footprint-based ground-truth links from Section 3.2. For each visit pair, the location-based distance threshold is set to the 95th percentile of distances between footprint-linked camera views. “Foot. Width” and “Dist. Thres.” give the average footprint width and the resulting distance threshold, respectively. “Foot. ALQ” and “Loc. ALQ” report the average number of links, i.e., ground-truth correspondences, per valid query under the two ground truths. R@10 and IR@10 denote Recall@K and IRRecall@K at K=10 , respectively.

Site	Visit years	Foot. width	Dist. thres	Foot. ALQ	Loc. ALQ	MegaLoc foot. links		MegaLoc Loc. links		NetVLAD foot. links		NetVLAD Loc. links	
						IR@10	R@10	IR@10	R@10	IR@10	R@10	IR@10	R@10
-	-	m	m	-	-	%	%	%	%	%	%	%	%
Site 1	2010–2011	1.65	1.37	15	18	9.8	59.5	8.7	60.5	3.1	29.1	2.9	31.2
	2010–2012	1.65	1.37	13	15	6.8	47.2	6.1	49.1	1.7	18.3	1.6	19.4
	2010–2013	1.64	1.37	15	17	5.0	36.3	4.5	37.8	1.8	20.9	1.8	22.7
	2011–2012	1.64	1.41	10	13	9.6	46.1	8.1	46.7	2.6	16.8	2.6	19.2
	2011–2013	1.63	1.36	11	13	6.7	33.9	6.1	35.2	2.9	18.5	2.7	19.6
	2012–2013	1.62	1.33	10	12	14.1	54.7	12.5	55.0	2.7	20.3	2.5	21.6
Site 2	2011–2012	1.82	1.99	14	26	4.5	32.4	3.4	42.0	1.6	16.9	1.5	26.1
	2011–2013	1.79	2.04	13	27	3.8	27.6	2.9	40.0	1.4	14.7	1.2	23.0
	2011–2017	1.95	2.32	16	35	2.4	22.7	2.1	36.4	1.2	14.4	1.1	21.7
	2012–2013	1.69	1.65	11	18	5.5	29.0	4.8	33.4	1.9	16.7	1.7	21.1
	2012–2017	1.86	2.18	12	27	3.5	23.9	2.7	32.0	1.5	13.9	1.4	22.5
	2013–2017	1.83	2.25	11	28	5.5	30.8	3.9	41.8	2.3	20.0	1.9	30.9
Site 3	2010–2012	1.64	1.47	12	16	8.6	49.3	6.6	51.3	1.2	12.3	1.0	13.7
	2010–2014	1.71	1.54	13	17	5.5	37.2	4.6	39.4	0.7	9.3	0.7	10.9
	2012–2014	1.67	1.56	11	15	8.7	49.4	6.8	51.1	1.4	13.4	1.3	15.3
Site 4	2009–2011	2.26	2.48	35	63	4.2	56.2	2.6	60.5	1.0	21.2	0.7	25.6
	2009–2013	2.07	2.46	31	57	4.3	47.9	2.3	51.4	0.8	14.9	0.5	19.7
	2011–2013	1.82	1.97	15	25	4.9	31.9	3.2	35.3	1.3	11.7	1.1	17.1
Site 5	2010–2012	1.82	1.87	16	27	3.6	28.6	2.6	32.8	1.0	13.0	0.9	17.6
	2010–2013	1.87	1.85	17	26	4.0	31.9	3.2	37.0	1.1	14.4	0.9	18.5
	2012–2013	1.88	1.95	24	39	4.7	42.2	3.0	46.1	1.1	19.0	1.0	24.4

For R@10, both models achieve consistently higher scores under the location-based than under the footprint-based ground truth, whereas IR@10 is generally lower under the location-based ground truth, consistent with the patterns observed in Figure 11. Table 4 further shows that visit pairs with the largest increase in R@10 when switching from footprint-based to location-based ground truth typically correspond to cases where the distance threshold is substantially larger than the average footprint width. This pattern is most evident at Site 2. For the 2012–2013 visit pair at Site 2, MegaLoc’s R@10 increases only modestly from 29.0% to 33.4% when moving from footprint-based to location-based ground truth, and the distance threshold (1.65 m) is very close to the average footprint width (1.69 m). In contrast, for the 2013–2017 visit pair, the distance threshold (2.25 m) exceeds the average footprint width (1.83 m) by a larger margin, and MegaLoc’s R@10 increases more substantially, from 30.8% to 41.8%. A similar pattern is observed for NetVLAD, with lower absolute R@10 values. In line with the IR@10 patterns, the additional location-based links introduced when the distance threshold substantially exceeds the footprint width tend to reduce IR@10, particularly for MegaLoc.

## 4 Discussion

### 4.1 Geometric registration

Across all reference sites, the geometric registration between visits achieves sub-decimeter alignment, with occasional local errors up to about 0.16 m that are not representative of the overall registration quality (see Section 3.1). The largest errors arise mainly from structural habitat changes in dense coral reef areas at Sites 1 and 2, where genuine 3D differences appear as local registration errors, and from missing overlap or reconstruction holes, where the registration algorithm may associate invalid point correspondences that inflate local error metrics without reflecting alignment quality where overlap is present. We also observe mild low-frequency warping in some reconstructions, likely caused by accumulated calibration and pose-estimation errors. For the approximately 35×35 m reference sites considered here, this typically contributes only a few centimeters of error, but larger sites might require non-rigid registration to align visits (Monji-Azad et al., 2023; Wang et al., 2022). For comparison, the smallest and largest average footprint widths for single visits in the dataset is approximately 1.62 and 2.59 m (corresponding to 2.0 and 3.2 m altitude in Table 2), respectively. This means that the most extreme 99th percentile registration errors are less than 10% of the smallest average image footprint width across the dataset, which we consider acceptable for the purpose of visual localization.

Overall, the largest registration errors can be traced to local structural changes, areas of missing overlap, or mild global warping rather than to systematic misalignment between visits. Consequently, the reported sub-decimeter error range is a more representative measure of the achieved alignment quality, and this level of consistency is sufficient to treat repeated surveys as sharing a common reference frame, enabling validation of long-term visual localization results at sub-decimeter accuracy.

### 4.2 Linking camera views with overlapping image footprints

Recent work defines VPR as the ability to recognize one’s location based on reference and query observations perceived from overlapping fields of view, implying that successful matches require a certain degree of visual overlap between observations (Garg et al., 2021). In this view, our footprint-based ground truth directly encodes this notion of place by linking camera views only when their estimated seafloor footprints overlap.

The results in Section 3.2 demonstrate that the footprint estimation and overlap criterion reliably establish cross-visit links between camera views, supporting the robustness of our footprint-based linking approach under varying geometry. The lower footprint IoU threshold used in this work $\left(\tau_{f}=0.07\right)$ is a deliberately conservative choice. It is based on the largest observed registration errors, which, as discussed in Section 4.1, are not representative of typical misalignment between visits. As a result, some camera view pairs with minimal but non-negligible visual overlap are likely excluded. However, this conservative threshold ensures that retained links exhibit meaningful visual overlap while minimizing false matches due to residual registration errors.

Across the sites and visit pairs, the relative camera–seafloor geometry is strongly affected by terrain structure and vehicle motion. Two dominant contributors are terrain ruggedness and vehicle altitude relative to the seafloor. Rugged terrain can limit the effective field of view and cause the image footprint to change substantially between neighboring views, so visual overlap becomes highly sensitive to local camera–seafloor geometry (see Figure 5, Panel (A)). This is the case at Site 4, which covers a boulder reef where images can exhibit range variations of several meters within a single field of view (see Figure 4). Local vertical seafloor structure can also interact with the vehicle’s altitude control and obstacle avoidance to produce distinct high- and low-altitude image sets (see Figure 5, Panel (B)). This occurs, for example, at Site 2, where a dense coral reef is elevated 6–8 m above the surrounding sediment plain, and at Site 5, where vertical rock cliffs of 2–3 m are present. In both cases, a single global distance threshold is either overly restrictive or overly permissive, because spatial proximity alone is a poor proxy for visual overlap.

Our approach of estimating 3D image footprints from range maps and linking views based on footprint overlap offers several advantages for ground-truthing visual localization results for near-nadir underwater imagery, because it explicitly accounts for vehicle attitude, altitude, and local terrain structure. The method handles cases where range varies across the field of view, unlike footprint models that use a single range and assume a flat seafloor (Eustice et al., 2008; Mahon et al., 2008). Because the linking criterion in Equation 11 operates directly on footprints and their IoU, it compensates for variations in relative geometry between views. The main drawback is the added complexity of requiring calibrated cameras, precise relative 3D poses, and reliable range estimates. In this work, range maps are derived by fusing stereo-based metric estimates with monocular relative estimates. Since the accuracy of the stereo component degrades with range, this limits the method to shorter operating ranges, depending on seafloor texture, water clarity, and imaging setup. In addition to range uncertainty, errors in camera attitude and horizontal position induce horizontal shifts of the footprint vertices that grow with range and off-axis angle, making the method more sensitive to pose errors at larger operating ranges.

For this dataset, the footprint-based ground-truthing approach yields a conservative ground truth with relatively few links per query. Nonetheless, by combining a principled footprint-based linking criterion with a deliberately conservative IoU threshold, we are confident that linked camera views exhibit meaningful visual overlap despite residual registration errors and varying camera–seafloor geometry, making the resulting ground truth suitable for benchmarking long-term visual localization algorithms. Future work could refine the footprint estimates by sampling more image points in Equation 7 to obtain more detailed 3D footprint polygons, or by using more advanced range map estimation methods (Ganj et al., 2025; Zhang et al., 2025).

### 4.3 Evaluating long-term visual place recognition performance

The results in Section 3.3.1 show that the Recall@K of state-of-the-art VPR models is significantly lower on the SEALOC dataset than on comparable terrestrial benchmarks (Ali-bey et al., 2023; Izquierdo and Civera, 2024; Berton and Masone, 2025), highlighting the challenges of long-term VPR in benthic environments. Performance varies substantially across sites, with higher recall for Sites 1, 3, and 4, which are dominated by more textured seafloor types. MegaLoc is the strongest model on the SEALOC dataset, with AnyLoc second, and the ViT-based models (AnyLoc, CliqueMining, MegaLoc, SALAD) consistently outperform the CNN-based models (CosPlace, EigenPlaces, MixVPR, NetVLAD).

For comparison with other underwater benchmarks, we consider the Eiffel Tower dataset, which is currently the only other curated dataset for long-term visual localization in benthic environments (Boittiaux et al., 2023). VPR performance on Eiffel Tower has been reported for AnyLoc, CosPlace, MegaLoc, MixVPR, and NetVLAD (Gorry et al., 2025). Across most of these models, Recall@1 and Recall@10 are lower on the SEALOC dataset than on Eiffel Tower. For example, MegaLoc is reported to achieve 33.7% Recall@1% and 65.1% Recall@10 on Eiffel Tower, compared to 21.9% and 46.3% at best on our sites (Gorry et al., 2025). In contrast to Eiffel Tower, the SEALOC dataset covers multiple sites with varied seafloor types and photic-zone benthic habitats, which exhibit stronger temporal dynamics and biological variability (Wong et al., 2023; Perkins et al., 2025; Glover et al., 2010). Taken together, the consistently lower Recall@K scores and the pronounced cross-site variability indicate that our benchmark exposes a more diverse and generally harder set of long-term VPR and visual localization challenges.

However, aggregate metrics obscure how performance depends on local seafloor features within each site. Visualizing the spatial distribution of successful and unsuccessful place recognitions for individual visit pairs (Figures 8, 9) provides additional insight into how seafloor characteristics and motion patterns shape VPR behavior. Across sites, successful place recognitions are not uniformly distributed but occur in spatial clusters that coincide with seafloor patches containing visually distinctive and persistent features across visits (for example, dense coral cover, rock–sand interfaces, or boulders with unique geometry) and with trajectory segments with sufficient visual overlap between query and database images. In contrast, visually homogeneous areas with limited persistent features, such as extensive soft-sediment plains, contain fewer and more sporadically distributed clusters of successful recognitions, although sporadic colonies of sessile organisms (for example, seagrass meadows) can still provide enough visual cues to recognize places.

Site-level differences in Recall@K (Table 3) therefore mainly reflect the availability and spatial distribution of such “good” regions for VPR. At Site 1, clusters of recognized queries occur frequently and are relatively evenly distributed over the dense coral reef, leading to higher mean recall, with similar behavior at Sites 3 and 4 over textured seafloors. At Site 2, the sharp contrast between coral reef, transition zones with sparse corals, and soft-sediment bottom produces an uneven pattern, where recognized queries concentrate over coral colonies and other localized features and are rare over homogeneous sediment, lowering overall Recall@K despite some locally good performance. This suggests that, in benthic environments, reliable long-term single-image VPR is inherently restricted to localized parts of each habitat where seafloor appearance is distinctive. Sufficient visual overlap between query and database images then becomes a second necessary condition for robust performance.

In Section 3.3.3, we reported how Recall@10 changes with revisit interval. Overall, Recall@10 decreases with increasing revisit interval, consistent with growing scene change between visits, with Site 1 providing the clearest evidence for temporal trends. For both Site 1 and Site 2, Figure 10 shows a marked drop in Recall@10 when the revisit interval increases from 1 to 2 years, followed by a more gradual decline that appears to approach a plateau at longer intervals. This supports the view that most of the visually relevant scene change for VPR occurs within the first few years after acquisition, after which additional change has a smaller marginal effect on performance. However, only Sites 1 and 2 have four visits and Site 2 has irregular revisit intervals, and visit-to-visit differences in coverage and altitude (Table 2) confound the interpretation, so the observed degradation should be viewed qualitatively rather than as a precise estimate. Despite these limitations, our results indicate that single-image VPR is most reliable for relatively short revisit intervals on the order of 1–2 years, and that longer intervals rapidly decrease performance, which has direct consequences for designing long-term monitoring campaigns and revisit schedules. To improve upon the analysis we recommend future work to only use images from seafloor patches that have been observed across all visits. This ensures that performance is evaluated on the same seafloor patches across visits and limits the effect of varying coverage between the different visit pairs.

Qualitative inspection of the query–database image pairs for Sites 1 and 2 (see Supplementary Materials) suggests several mechanisms that contribute to this temporal degradation of VPR performance. On the biological side, growth, mortality, and overgrowth of corals and associated taxa change occlusion relationships, edge geometry, and fine-scale texture. Plate and branching corals grow to hide previously visible colonies, while growth and movement of seaweed and other macroalgae partially obscure underlying structures and substrate. Bleached or dead colonies are replaced or overgrown by algae and encrusting taxa, and pigmentation changes alter colors even where the gross 3D structure remains similar. On sediment plains and in nearby areas with partial sediment cover, scouring and local redistribution of sand partially or fully bury small rocks and coral rubble, hiding small-scale detail and altering the geometry of sediment–rock interfaces and edges. Together, these processes progressively change the appearance and distinctiveness of seafloor patches that VPR models rely on. The same qualitative examples also show that successful matches can be obtained despite substantial differences in vehicle heading and moderate differences in altitude between the query and database images. In several pairs, in-plane rotations and scale changes are evident, yet retrieved images still share recognizable seafloor features. This suggests that the strongest VPR models are reasonably tolerant to rotation and scale variation as long as there is sufficient visual overlap, whereas horizontal displacement between the images and the associated loss of common visual seafloor features is likely the more critical pose-related factor for successful VPR.

While the mean Recall@K reported in Section 3.3.1 is useful to compare model performance, practical VPR deployment on underwater robots must account for computational and memory constraints. ViT-based models generally require more computation than CNN-based models (Cordonnier et al., 2020). Within this group, AnyLoc is particularly expensive, producing 49,152-dimensional descriptors, whereas CliqueMining, MegaLoc, and SALAD use 8,448-dimensional descriptors, resulting in a substantially smaller database footprint and faster retrieval speeds. Considering both retrieval performance and resource usage, MegaLoc is a reasonable default choice for systems that can support ViT-based models, whereas EigenPlaces offers the best trade-off among the CNN-based methods for platforms that cannot accommodate transformer-based architectures. A table with an overview of the image descriptor dimensions, typical descriptor database memory footprints, and average inference speeds is provided in Supplementary Materials.

Our study has several limitations that qualify these findings. Constructing long-term benthic datasets with precisely registered imagery is labor-intensive, which constrains both the number of sites and the diversity of motion patterns and habitat types we can cover. The temporal analysis is dominated by two sites and includes irregular revisit intervals, so the observed degradation over time should be interpreted qualitatively rather than as a precise estimate of performance loss. In addition, we only evaluate single-image, descriptor-based VPR pipelines without explicit map building or uncertainty estimation, whereas deployed systems will typically combine retrieval with additional localization and fusion modules.

To improve long-term visual localization performance in benthic environments, future work could exploit our observations of clustered successful recognitions by exploring map representations that decompose habitats into sub-maps or place representations formed from spatially and visually coherent image clusters (Li et al., 2015; Steinberg et al., 2010; 2015; Liang et al., 2025). Such map representations could reduce the effective search space during retrieval, expose which visual features support successful localization, and incorporate priors on the expected persistence of appearance at each place (Johns and Yang, 2011). Another promising direction is to use information from multiple query images along trajectory segments (Stenborg et al., 2020; Toft et al., 2022), increasing the spatial context used for localization and reducing reliance on high overlap between individual query–database image pairs. Accurate short-term odometry from dead-reckoning navigation sensors (Fogh Sørensen et al., 2025) can provide relative pose estimates between query images, which can then be used to geometrically reject or verify VPR retrievals (Claxton et al., 2024).

### 4.4 Evaluating differences in ground-truthing strategies

The comparison between footprint-based and location-based ground-truthing strategies in Section 3.4 shows that VPR performance metrics are sensitive to how “relevance” is defined. The location-based ground truth increases the average number of links per query (ALQ), i.e., the average number of ground-truth correspondences, relative to the footprint-based definition, and because our VPR recall R@K is non-decreasing as additional ground-truth links are added, switching to the location-based ground truth systematically raises R@10. This can give an overly optimistic impression of performance by rewarding any retrieval within a generous distance radius, and similar effects have been reported for dense underwater datasets where overly permissive thresholds allow random baselines to approach SOTA Recall@K at moderate rank cutoffs (K≳10) (Gorry et al., 2025). In contrast, the information-retrieval definition IR@K normalizes by the total number of relevant links and therefore decreases when the ground truth is expanded but many of the additional links are not retrieved, as observed in our experiments. The larger drop in IR@10 for the stronger VPR models in Figure 11 indicates that these models expose inconsistencies between the ground-truth definitions more clearly.

As discussed in Section 4.2, terrain relief and vehicle altitude variations at Sites 2, 4, and 5 cause substantial differences between footprint-based and location-based ground truths because spatial proximity is a poor proxy for visual overlap in these settings. At sites with little relief and relatively constant altitude, such as Site 1, the effective image footprint width is approximately constant, so the location-based ground truth can accurately approximate the footprint-based ground truth, and the two definitions yield similar ALQ values with only small changes in R@10 and IR@10. In contrast, at sites with strong vertical transitions or highly rugged terrain, a relatively loose distance threshold can include many view pairs with limited or no visual overlap, inflating the number of links and amplifying the discrepancy between R@10 and IR@10. Under these conditions, a single global distance threshold cannot capture geometric variations between views and will be overly restrictive in some cases and overly permissive in others.

These findings suggest that overlap-aware ground truths are preferable to purely distance-based criteria for densely mapped near-nadir imagery, since global distance thresholds cannot account for terrain relief, ruggedness, or variations in vehicle altitude. They also show that the VPR definition of recall, R@K, is inherently favorable to permissive ground-truth definitions, and that additional metrics such as the information-retrieval definition of recall or mean average precision (Garg et al., 2021) should be reported to provide more nuanced insight that can be aligned with task- and domain-specific requirements. A limitation of our study is that we evaluate only a single location-based distance threshold per visit pair when comparing the ground-truthing strategies. Our supplementary analysis shows that the corresponding location-based metrics are sensitive to this choice, although the qualitative trends under more permissive thresholds remain unchanged.

Building on these observations, a natural alternative to a footprint-based ground truth is a location-based ground truth with an adaptive distance threshold. In such a scheme, the threshold between camera views can be set from an estimate of the effective image footprint size, for example, using a flat-seafloor model together with altitude readings from a DVL or altimeter. This can partially compensate for altitude-induced changes in footprint size and would likely reduce discrepancies between location-based and footprint-based metrics at sites with non-rugged terrain. However, because the threshold is still defined purely in terms of the distance between views, it cannot account for occlusions, so nearby views that see different parts of the seafloor will still be labeled as relevant. Site 4 in the SEALOC dataset illustrates this directly as large boulders with several meters of vertical relief occlude different portions of the surrounding seafloor depending on the camera’s position, such that spatially proximate views may observe largely non-overlapping seafloor patches. In our setting, we therefore view such adaptive thresholds as a useful baseline when detailed range data are unavailable, but not a replacement for overlap-aware ground truths for rugged terrain.

## 5 Conclusion

In this work, we addressed the lack of curated datasets for long-term visual localization in benthic environments and the lack of ground-truthing methods suited for near-nadir underwater imagery. We presented SEALOC, a curated AUV imagery dataset including raw and color-corrected stereo imagery, camera calibrations, and camera poses registered to sub-decimeter accuracy from revisits to five benthic reference sites. To our knowledge, this is the first curated underwater dataset for long-term visual localization that covered photic-zone habitats across multiple sites with diverse seafloor characteristics. Building on this dataset, we proposed a footprint-based ground-truthing method for near-nadir imagery that estimates 3D image footprints and links camera views whose footprints overlap, accounting for terrain relief and vehicle altitude variations and yielding conservative but visually meaningful ground-truth links.

Using this footprint-based ground truth, we benchmarked eight state-of-the-art visual place recognition (VPR) methods and found that recall on the SEALOC dataset was substantially lower than on terrestrial and existing underwater benchmarks, indicating that the sites in the dataset present a diverse and harder set of challenges. Performance varied strongly across and within sites, with successful recognitions clustering in regions with distinctive, persistent seafloor features. Comparing our footprint-based ground truth to a traditional location-based ground truth showed that spatial proximity alone could systematically overestimate Recall@K, especially at sites with rugged terrain or large altitude variations, whereas the footprint-based ground truth was more aligned with actual VPR retrievals.

These findings highlight that accurate ground-truthing of visual localization for near-nadir underwater imagery requires precise geometric information and overlap-aware definitions of “place”, and that reliable long-term visual localization in benthic environments will likely require more sophisticated approaches than single-image VPR alone for reliable performance.

## Data Availability

The SEALOC dataset can be found in the NIRD Research Data Archive: (https://doi.org/10.11582/2026.qro1lf3z). Our code for using the SEALOC dataset will be made public upon publication: https://github.com/sealoc/sealoc.
