# Supplementary material for: Long-term visual localization in dynamic benthic environments: the SEALOC dataset, footprint-based ground truth, and visual place recognition benchmark
Source: Front Robot AI. 2026 Jun 8;13:1821019. doi: 10.3389/frobt.2026.1821019 (PMC13284073; doi:10.3389/frobt.2026.1821019)
Supplement: Supplementary file 1 [file DataSheet1.pdf]

Supplementary Material

S1 SENSOR SUITE AND TECHNICAL SPECIFICATIONS

S1.1 Sensor Suite

Table S1 shows an overview of the selected subset of the sensor suite of AUV Sirius across different years. The description of the AUV Sirius sensor suite were compiled from previously published work [1][2][3]and campaign reports [4]. For a more detailed overview of the sensor suite of AUV *Sirius* we refer the reader to these references.

Year	Category	Sensor Suite
2009 – 2011	Camera	AVT Prosilica GC1380 12bit 1360×1024 CCD stereo
	Light Sources	2x 4J Xenon flashes
	Navigation	u-blox GNSS Receiver
	Navigation	Paroscientific Digiquartz Pressure Sensor
	Navigation	Teledyne RDI Work Horse Navigator DVL
	Navigation	LinkQuest TrackLink 1500HA USBL
	Environmental	Seabird SBE 37-SIP CTD
	Environmental	WET Labs ECO Puck Triplet Sensor
2012 – 2014	Camera	AVT Prosilica GC1380 12bit 1360×1024 CCD stereo
	Light Sources	2x LED strobes, 30000 lm each
	Navigation	u-blox GNSS Receiver
	Navigation	Paroscientific Digiquartz Pressure Sensor
	Navigation	Teledyne RDI Work Horse Navigator DVL
	Navigation	LinkQuest TrackLink 1500HA USBL
	Environmental	Seabird SBE 37-SIP CTD
	Environmental	WET Labs ECO Puck Triplet Sensor
2017	Camera	AVT Prosilica GC1380 12bit 1360×1024 CCD stereo
	Light Sources	2x LED strobes, 30000 lm each
	Navigation	u-blox GNSS Receiver
	Navigation	Paroscientific Digiquartz Pressure Sensor
	Navigation	Teledyne RDI Work Horse Navigator DVL
	Navigation	Evologics S2C R 18/34 USBL
	Environmental	Seabird SBE 37-SIP CTD
	Environmental	WET Labs ECO Puck Triplet Sensor

Table S1. Overview of a selected subset of sensor suite of AUV *Sirius* for the different years in our dataset.

[1] ”Monitoring of Benthic Reference Sites”, Williams et al. (2012), DOI: https://doi.org/10.1109/MRA.2011.2181772.

[2] ”Benthic monitoring with robotic platforms - the experience of Australia”, Pizarro et al. (2012), DOI: https://doi.org/10.1109/UT.2013.6519909.

[3] ”Hyperspectral Benthic Mapping From Underwater Robotic Platforms”, Bongiorno, Daniel L. (2014), DOI: http://hdl.handle.net/2123/13024

[4] https://researchdata.edu.au/imos-autonomous-underwater-auv-sirius/476685

S1.2 Technical Sensor Specifications

Table S2 shows the technical specifications for a selected subset of AUV *Sirius*' sensors. The technical specifications were compiled from technical datasheets provided by the manufacturers and vendors [5][6][7][8][9].

Sensor	Specifications
AVT Prosilica GC1380	Bit depth: 12 bit<br>Resolution: 1360 × 1024 pixels (1.4 Megapixel)<br>Sensor: Sony ICX285 EXview HAD CCD<br>Sensor size: Type 2/3"<br>Pixel size: 6.45 μm × 6.45 μm
Paroscientific Digiquartz Pressure Sensor	Typical accuracy: 0.01% of full scale<br>Pressure / depth ranges: 0–700 m water depth
Teledyne RDI Work Horse Navigator DVL	Frequency: 1200 kHz<br>Configuration: 4-beam Janus array, convex, 30° beam angle<br>Velocity range (bottom track): ±10 m/s<br>Single-ping precision at 1 m/s: ±0.3 cm/s<br>Minimum altitude: 0.5 m<br>Maximum altitude: 30 m<br>Velocity resolution: 0.1 cm/s<br>Bottom-track accuracy (typical): ±0.3% ± 0.2 cm/s<br>Ping rate: up to 7 Hz<br>Altitude measurement: single-ping bottom altitude per beam<br>Integrated sensors: fluxgate compass, pitch and roll, temperature
LinkQuest TrackLink 1500HA USBL	Operating frequency: 31.0–43.2 kHz<br>Slant range accuracy: 0.20 m<br>Transponder maximum depth: up to 1500 m<br>Working range with ship noise: up to 1000 m<br>Positioning accuracy: 0.25° (better than 0.5% of slant range)<br>Operating beamwidth: 120–150°
Evologics S2C R 18/34 USBL	Operating frequency: 18–34 kHz<br>Slant range precision: 0.01 m<br>Depth rating (metal/OEM versions): 2000 m<br>Typical operating range: about 2000 m (mid-range applications)<br>Communication type: full-duplex underwater acoustic modem<br>Beam pattern: horizontally omnidirectional (donut-shaped)

Table S2. Technical specifications for the camera, and main navigation sensors for AUV Sirius.

[5] https://www.gosubsea.no/product/teledyne-rdi-work-horse-navigator-1200-khz-600khz-300khz-dvl
[6] https://www.link-quest.com/html/1500ha_sys_spec.htm
[7] https://www.evologics.com/underwater-acoustic-modem/pro-18-34
[8] https://paroscientific.com/pdf/D50_Series_8000.pdf
[9] https://www.altavision.com.br/Arquivos/AVT/Manuals/GC1380_User_Manual.pdf

S2 DATA PROCESSING PARAMETERS AND CONFIGURATIONS

S2.1 Image Color Correction Parameters

Table S3 shows the parameters for the image color correction for the RGB and monochrome imagery using the multi-image grayworld algorithm.

Parameters	RGB Camera	Monochrome Camera
Batch size	200	200
Desired Channel Mean	[0.35, 0.35, 0.35]	0.35
Desired Channel Std. Dev.	[0.12, 0.12, 0.12]	0.12

Table S3. Parameters used for the image color correction for the RGB and monochrome camera. The same parameters have been used across all visits in the dataset.

S2.2 Geometric Registration Parameters

Table S4 shows the configurations for the geometric registration pipeline used to register the visits for each reference site.

Registration Stage	Estimation Method	Parameter	Configuration 1	Configuration 2
Stage 1	FPFH-RANSAC	Voxel Size	0.08 m	0.20 m
		Distance Threshold	0.06 m	0.15 m
		Max. RANSAC Iterations	100 000 000	100 000 000
Stage 2	Colored ICP	Voxel Size	0.10 m	0.20 m
		Distance Threshold	0.15 m	0.20 m
		Colored ICP Geometry Weight	0.968	0.968
		Huber Kernel Weight	0.40	0.40
		Max. ICP Iterations	50	50
Stage 3	Colored ICP	Voxel Size	0.05 m	0.05 m
		Distance Threshold	0.05 m	0.05 m
		Colored ICP Geometry Weight	0.968	0.968
		Huber Kernel Weight	0.40	0.40
		Max. ICP Iterations	50	50
Stage 4	Colored ICP	Voxel Size	0.02 m	0.02 m
		Distance Threshold	0.02 m	0.02 m
		Colored ICP Geometry Weight	0.968	0.968
		Huber Kernel Weight	0.40	0.40
		Max. ICP Iterations	50	50

Table S4. Parameters for the two configurations of the geometric registration pipeline used to register the source visits to each reference site. Configuration 1 is used to register the visits for Site 1, while Configuration 2 is used to register the visits to Site 2, 3, 4, and 5. Overall, Configuration 1 performs registration at finer spatial resolutions than Configuration 2.

S3 COORDINATE REFERENCE FRAMES

Table S5 shows the origin of the NED (North-East-Down) coordinate frames that is used to convert coordinates from the local NED frame to the global frame on WGS84 (EPSG:4326).

Site	Longitude	Latitude	Height
-	deg	deg	m
Site 1	113.94675015	-28.81316413	-18
Site 2	114.01359028	-28.70846340	-35
Site 3	148.00901624	-43.13349486	-42
Site 4	148.34164114	-41.25298592	-28
Site 5	153.47810347	-27.13238831	-18

Table S5. Origin of the NED coordinate frame for the reference sites in the dataset.

S4 VISUAL PLACE RECOGNITION MEMORY AND INFERENCE SPEED

Table S6 shows the image descriptor size, the descriptor database size for a database of 3000 images, and the average inference (retrieval) speed for a single query image with 100 proposals. The experiments were performed on a computer with a 12th Gen Intel(R) Core(TM) i7-12700K CPU and a NVidia GeForce RTX 3080 Ti GPU. The average retrieval speed for models with the same image descriptor dimensions varies due to different workloads for the CPU during the experiments.

VPR Model	Image Descriptor Dimensions	Image Descriptors Memory Footprint (3000 images)	Average Inference Speed per Query (100 candidates per query)
-	-	Megabytes	ms
AnyLoc	49152	294.9	32.82
CliqueMining	8448	50.7	8.30
CosPlace	2048	12.3	2.36
EigenPlaces	2048	12.3	2.09
MegaLoc	8448	50.7	8.60
MixVPR	512	3.1	0.15
NetVLAD	4096	24.6	3.77
SALAD	8448	50.7	8.02

Table S6. Image descriptor size, image descriptor database sizes, and average inference speed per query. Database descriptors memory footprint are calculated assuming 16-bit floating point precision. The retrieval speeds are averaged over 3000 query images, where 100 candidates are retrieved for each query from a database of 3000 database images.

S5 RECOGNIZED QUERY-DATABASE IMAGE PAIRS

S5.1 Recognized Query-Database Image Pairs For Site 1 2010-2013

Figure S1 shows query-database image pairs recognized by MegaLoc from the 2010-2013 visit pair to Site 1. The image pairs provide qualitative examples of seafloor features that are recognized by the strongest VPR models, and the various scene changes that are observed across visits to the site, which covers a dense coral reef.

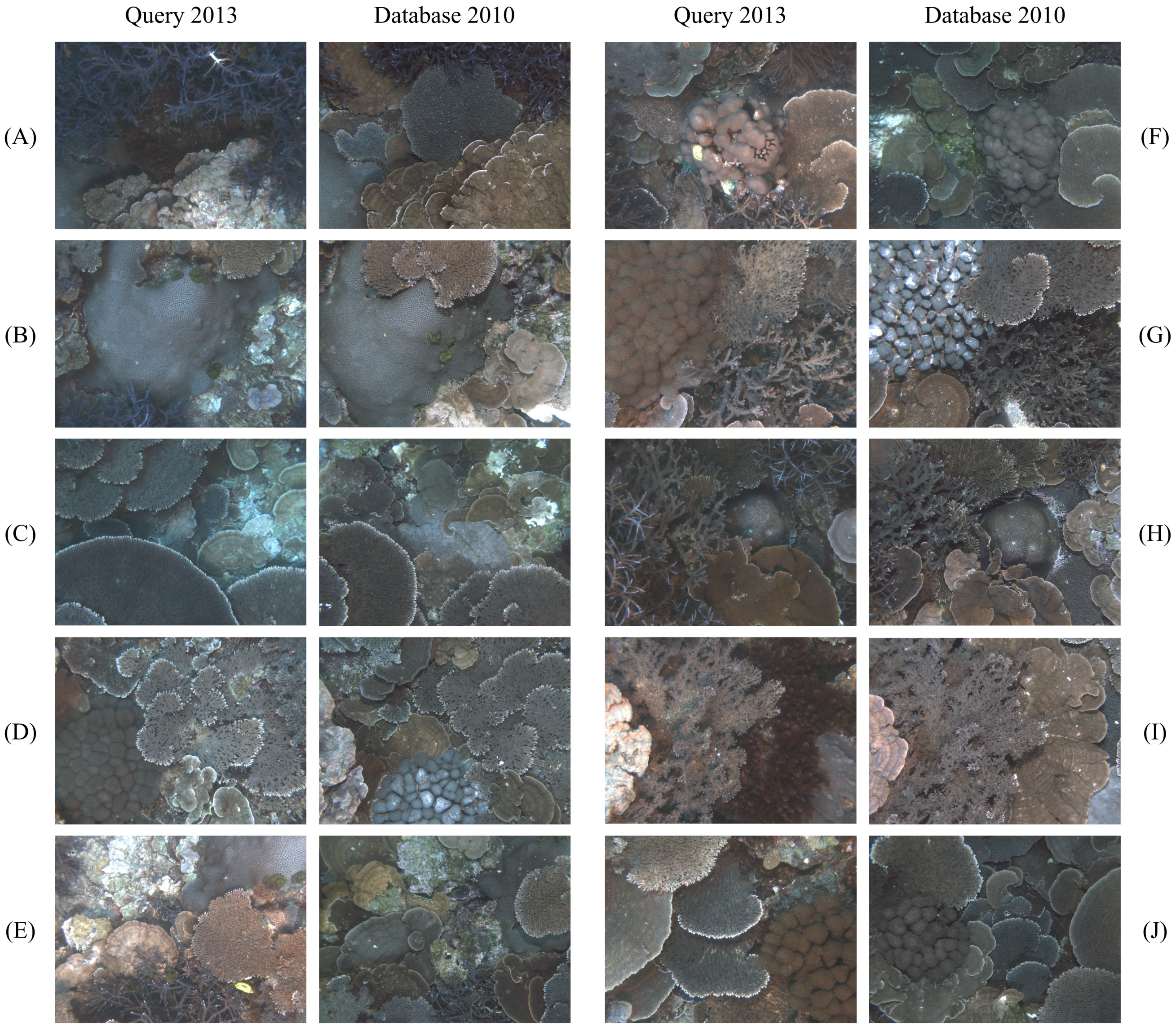

Figure S1. Examples of query-database image pairs successfully recognized by MegaLoc from Site 1 with 2010 database images retrieved for 2013 query images. The examples illustrate scene changes including among others growth of coral colonies, coral decay and pigmentation changes, and algae growth. Across the query-database image pairs one can typically recognized massive coral colonies or assemblages, which are believed to serve as ”anchors” providing enough visual cue for successful visual place recognition.

From the image pairs, we observe several types of scene change that alter the visual appearance of the same seafloor patch over the 3-year interval. Growth of plate and branching corals occludes previously

visible colonies (Panels A, B, C, E, H), coral decay and pigmentation changes modify local color and texture while leaving overall structure intact (Panels A, B, E, I), dead coral skeletons are progressively overgrown by algae and other encrusting taxa (Panel I), and changes in polyp extension and fine-scale relief of lobate corals, which affect apparent texture and color (Panel D, G, J).

The recognized query–database image pairs predominantly correspond to seafloor patches with high structural complexity and mixed coral morphologies. In these regions, large plate and massive colonies provide stable geometric anchors, while branching and soft corals, encrusting forms, and exposed substrate create distinctive constellations of edges, and textures. The combination of high 3D relief, and multi-scale texture makes these patches visually unique compared with flatter, more homogeneous areas such as sand or rubble, and they are repeatedly retrieved as successful matches across years. Even when biological change alters fine-scale appearance, the overall arrangement of major colonies remains sufficiently characteristic to support robust VPR. In addition, several recognized pairs exhibit noticeable differences in camera heading, resulting in in-plane rotations between the query and database views (Panels C and J). That these pairs are still matched suggests that the strongest VPR models maintain a degree of robustness to rotational changes when distinctive reef structure is present.

S5.2 Recognized Query-Database Image Pairs For Site 2 2011-2013

Figure S2 shows query–database image pairs recognized by MegaLoc for the 2011–2013 revisit of Site 2, which spans dense coral reef, sediment plain, and the transition zone between them. The pairs provide qualitative examples of the scene changes occurring over the revisit interval and the seafloor features that still provide sufficient visual cues for successful visual place recognition.

Across the image pairs we observe several types of scene change that directly affect the visual appearance of the same seafloor patch. Scouring and local redistribution of sediment partially or fully cover small rocks, coral rubble, and smaller colonies, altering edge structure and creating new homogeneous regions of bright sand (Panels A–D, J). Growth and movement of seaweed and other macroalgae introduce new dark, filamentous textures that partially occlude underlying colonies or the surrounding substrate (Panels C, E, F, G, J). In some cases, bleached or dead corals become overgrown by algae and encrusting taxa, producing marked changes to local community composition (Panel H). Finally, growth and pigmentation changes of live corals modify the composition of local assemblages and the apparent color and texture of individual colonies (Panels E, I, J).

Despite these changes, the recognized query–database pairs typically contain distinctive seafloor features that remain visually recognizable across visits. In the sediment-dominated areas, isolated but relatively large colonies or small clusters of colonies embedded in otherwise flat, homogeneous sediment act as stable geometric anchors (Panels A–D). In the reef and transition-zone, high-relief massive and plate colonies, and compact assemblages of corals and sponges, create characteristic constellations of edges and multi-scale textures (Panels E–J). This combination of stable large structures within otherwise simpler surroundings provides enough persistent, spatially unique visual cues to support robust VPR at Site 2. Notably, several recognized pairs exhibit clear differences in camera pose, with heading differences produce in-plane rotations between query and database views (Panels C, D, E, F, G), and altitude variations leading to scale differences (Panels C, G). The fact that MegaLoc still retrieves these pairs indicates that the strongest VPR models are reasonably robust to rotation and moderate scale changes, provided that distinctive seafloor structure is present.

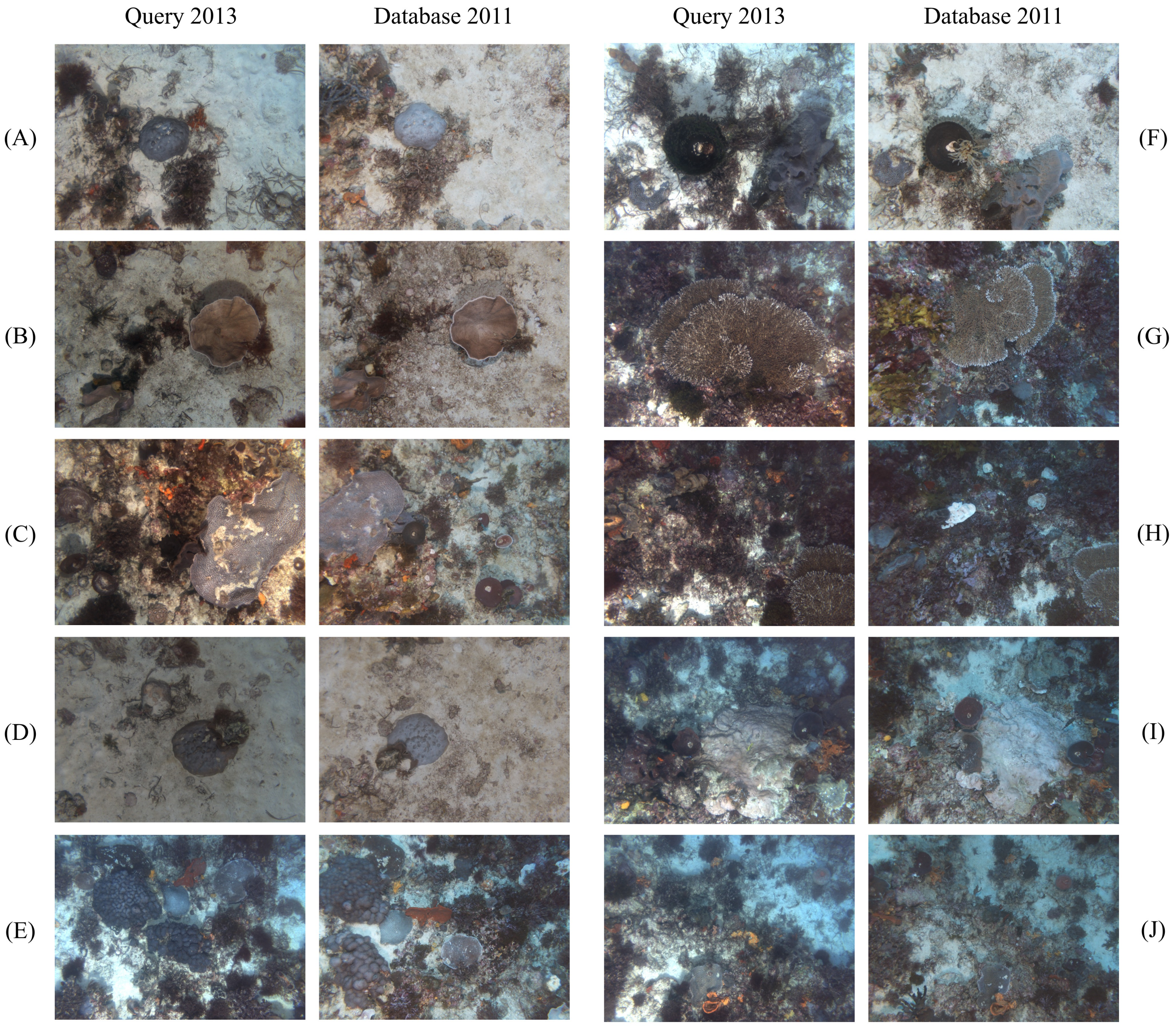

Figure S2. Examples of query-database image pairs successfully recognized by MegaLoc from Site 1 with 2011 database images retrieved for 2013 query images. The query-database image pair examples show scene changes for the dense coral reef, sediment plain, and transition zone. In the dense coral reef one can typically recognize larger, distinctive coral colonies across the query and database image. In the sediment plain individual coral colonies are recognizable, while smaller surrounding rocks and coral debris are covered by sediment.

S6 VISUAL PLACE RECOGNITION RESULTS

S6.1 Visual Place Recognition Results for Site 3 2010-2012

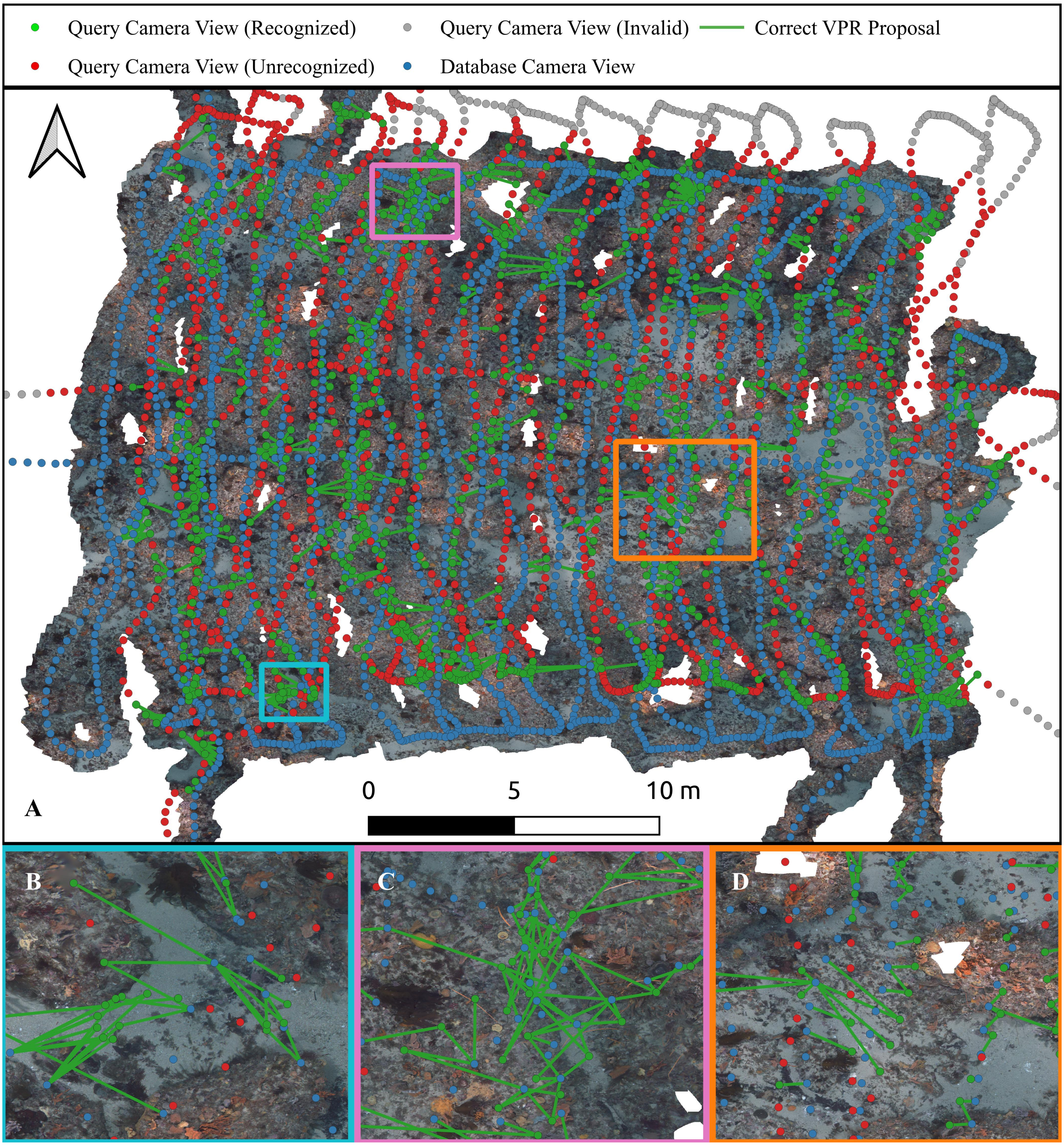

Figure S3. Visual place recognition results at Site 3 rendered on top of an orthomosaic derived from the 2010 images, using MegaLoc with $K = 5$ to retrieve 2010 database images for 2012 query images.

S6.2 Visual Place Recognition Results for Site 4 2009-2011

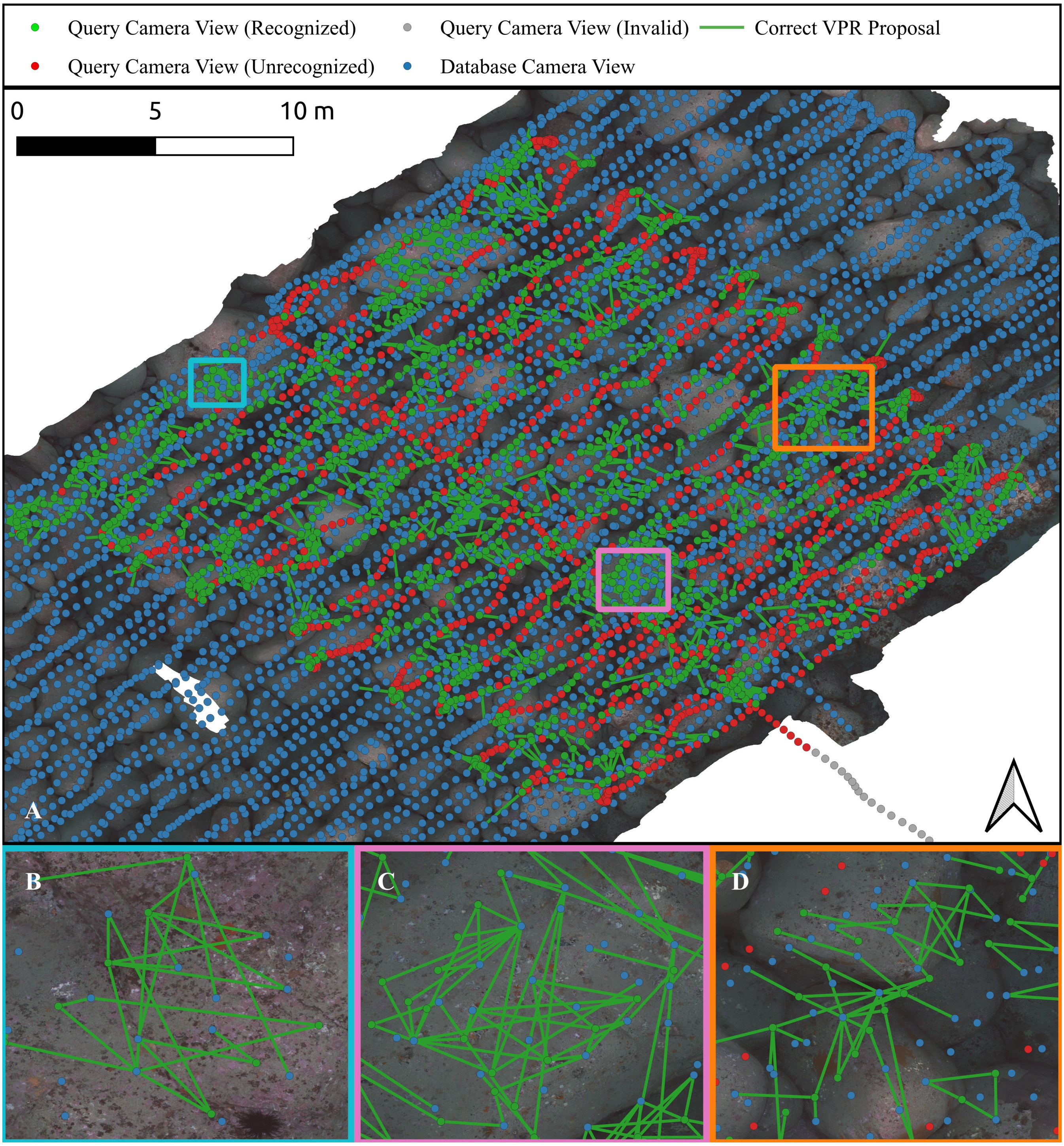

Figure S4. Visual place recognition results at Site 4 rendered on top of an orthomosaic derived from the 2009 images, using MegaLoc with $K = 5$ to retrieve 2009 database images for 2011 query images.

S6.3 Visual Place Recognition Results for Site 5 2010-2013

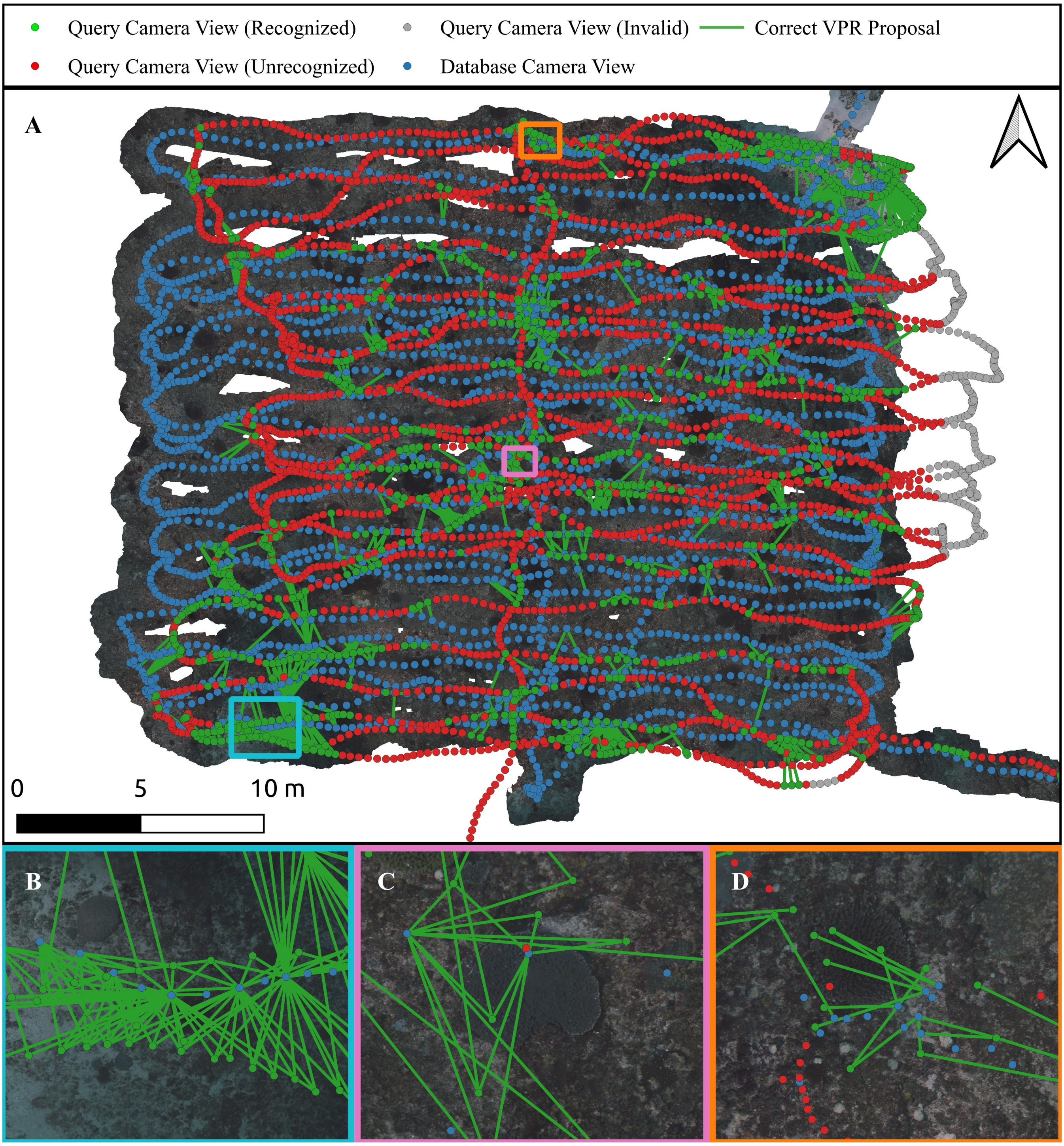

Figure S5. Visual place recognition results at Site 5 rendered on top of an orthomosaic derived from the 2010 images, using MegaLoc with $K = 5$ to retrieve 2010 database images for 2013 query images.

S7 VERY HARD, HARD, AND EASY QUERY IMAGES

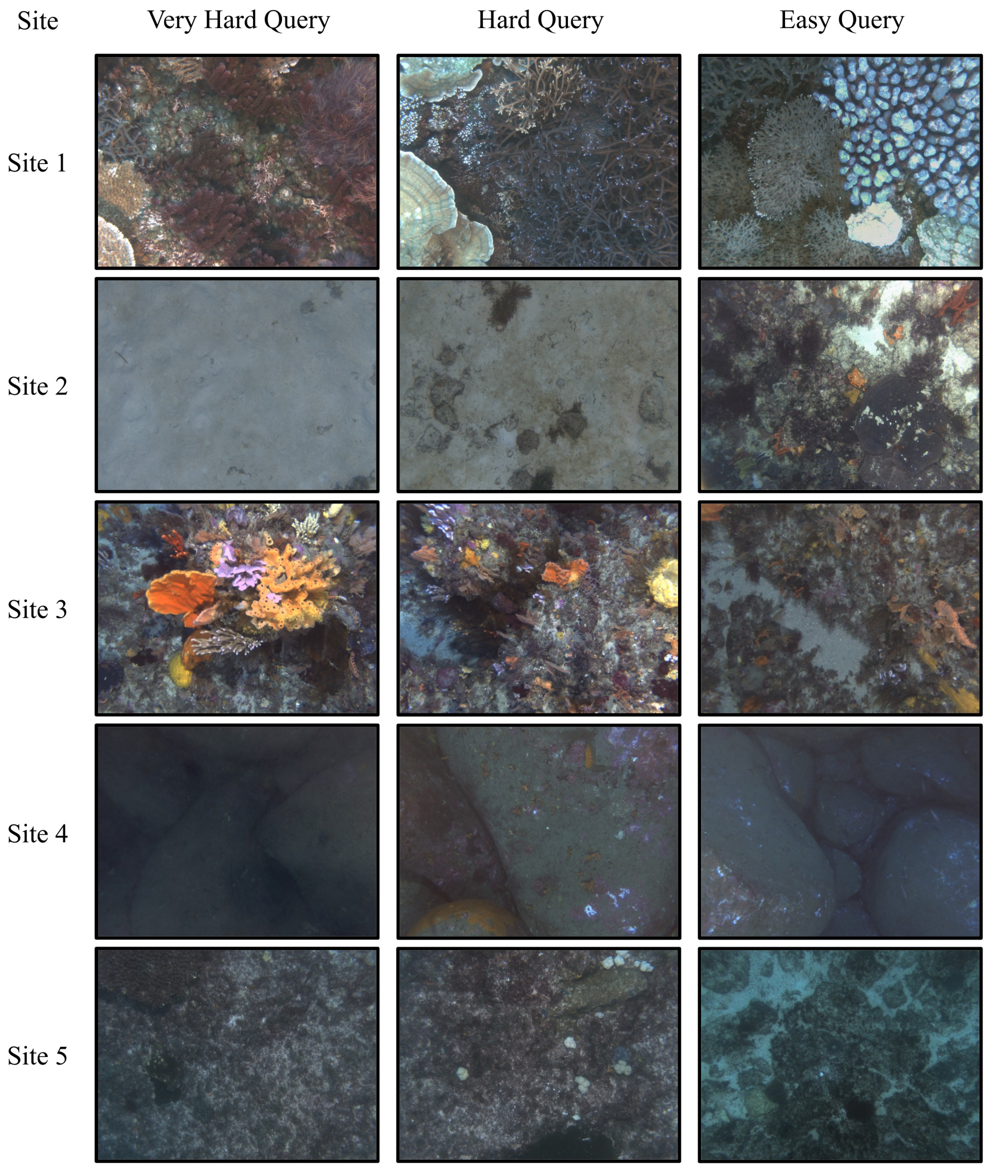

Figure S6. Examples of very hard, hard, and easy query images from each benthic reference site. The very hard queries are not recognized by any VPR model, hard queries are recognized by the best models, i.e. MegaLoc and AnyLoc, and easy queries are recognized by all models.

S8 VISUAL PLACE RECOGNITION PERFORMANCE METRICS FOR LOCATION-BASED GROUND TRUTH WITH VARYING DISTANCE THRESHOLD

Figure S7 shows Recall@10 and IRecall@10, denoted R@10 and IR@10, respectively, averaged over visit pairs for each site, as a function of the distance threshold used for the location-based ground truth.

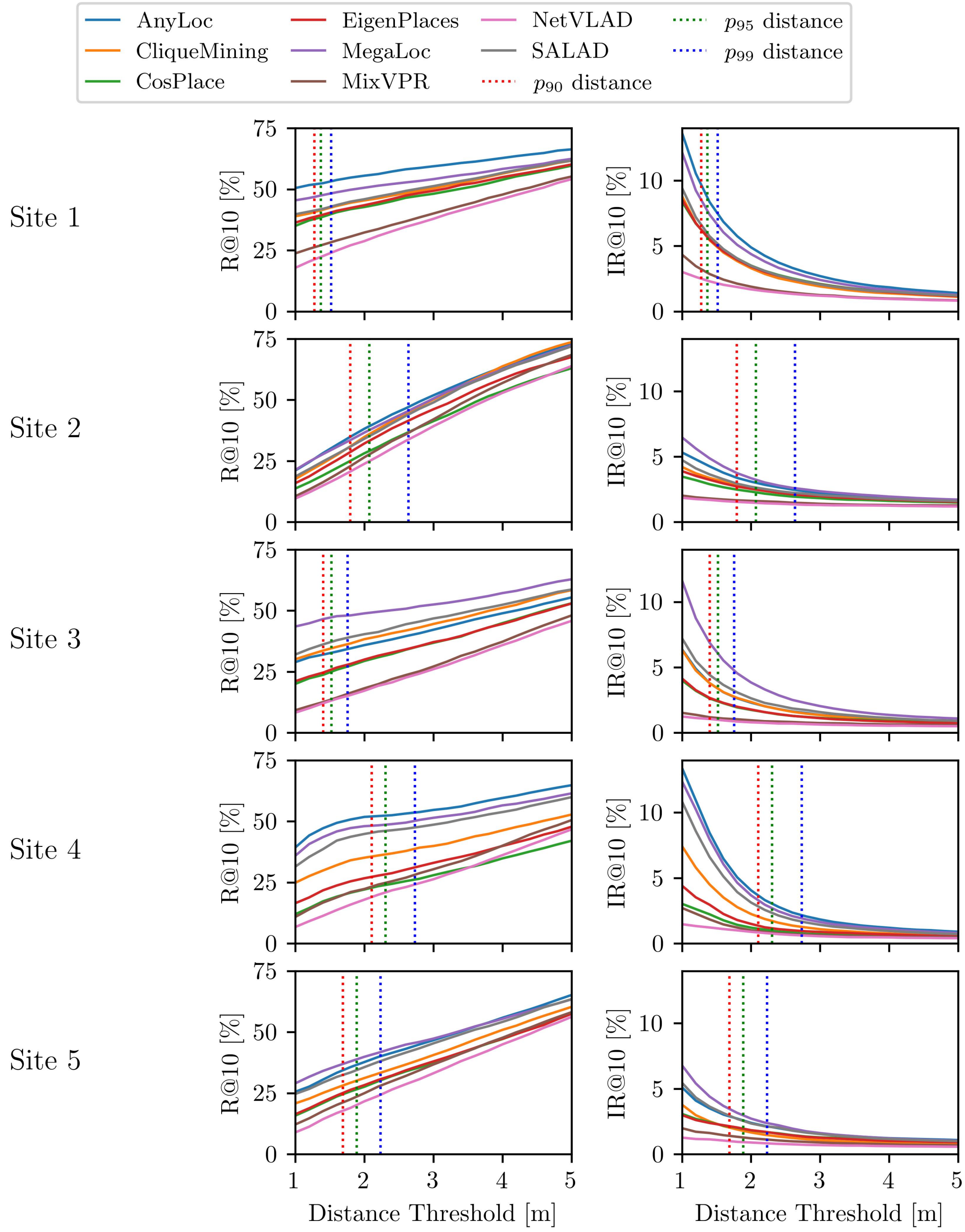

Figure S7. Visual place recognition performance under location-based ground truth with varying distance thresholds (in meters). Recall@K and IRecall@K with rank cutoff $K = 10$ are denoted R@10 and IR@10, respectively. p_{90}, p_{95} and p_{99} denotes the 90th, 95th, and 99th percentile distances between footprint links averaged over visit pairs for each site.

The general trends in Figure S7 match the observations from the comparison between footprint-based and location-based ground truth, with R@10 increasing and IR@10 decreasing for more permissive location-based thresholds. The magnitude of these changes varies across sites, indicating that the sensitivity of R@10 and IR@10 to the threshold depends on site-level differences in survey geometry and seafloor characteristics. For more permissive thresholds, performance gaps between stronger and weaker VPR models shrink in both R@10 and IR@10, suggesting that weaker models generally benefit disproportionately from looser ground-truth definitions. For Site 2 and Site 5, which are considered to be more challenging sites, the sensitivity in R@10 is more similar across the strong and weak VPR models.

S9 DATA ORGANIZATION AND FORMATS

The SEALOC dataset is organized into image archives and accompanying metadata describing camera-related data, geometry, and grouping. Table S7 summarizes the file patterns, formats, and contents.

Filename / Pattern	Format	Description
<SITE_GEOHASH>_<YYYYMMDD_HHmmSS>_images_raw.zip	ZIP + TIFF	Raw RGB (debayered) and monochrome stereo image pairs.
<SITE_GEOHASH>_<YYYYMMDD_HHmmSS>_images_grayworld.zip	ZIP + PNG	Color corrected stereo image pairs. The image pairs are corrected with the methodology outlined in the main manuscript.
`sealoc.db`	SQLite	Database with ORM models, including cameras, camera sensors, camera calibrations, camera poses, camera footprints, camera groups, and camera bundles.
`cameras.csv`<br>`camera_calibrations.csv`<br>`camera_groups.csv`<br>`camera_sensors.csv`<br>`camera_footprints.csv`<br>`camera_bundles.csv`<br>`camera_poses.csv`	CSV	CSV exports closely resembling the tables in `sealoc.db` for quick inspection and scripting.

Table S7. Overview of the SEALOC dataset file formats and descriptions.

The SQLite database `sealoc.db` defines an object–relational mapping (ORM) for the camera-related data models in the SEALOC dataset. Figure S8 shows an entity relationship diagram (ERD) for the ORM. The software library provided with the dataset maps these ORM models to the following domain models, which users of the dataset interact with programmatically:

- `camerasensor` Model for a physical camera (imaging) sensor with a given width and height. For multi-camera systems, a camera sensor can also have an assigned master camera sensor. The location and rotation of the camera sensor is defined relative to its master sensor. Camera sensor has a one-to-many relationship with camera.
- `cameracalibration` Model for the intrinsic calibration of a camera sensor. The calibration follows the OpenCV convention for the pinhole camera model.
- `camerapose` Model for a camera pose with attitude represented as yaw, pitch, and roll, and a 3D location point and a spatial reference system identifier (SRID).
- `camerafootprint` Model for a camera (image) footprint with 3D vertices defining the footprint polygon and a SRID defining the coordinate reference system for the polygon vertices.
- `camera` Model for a camera (view) with an associated image label and related sensor, group, pose, and footprint. For multi-camera systems, a camera can have an assigned master camera.
- `cameragroup` Model for a group of cameras and sensors.
- `camerabundle` Model for a camera bundle, i.e., an aggregate of camera groups.

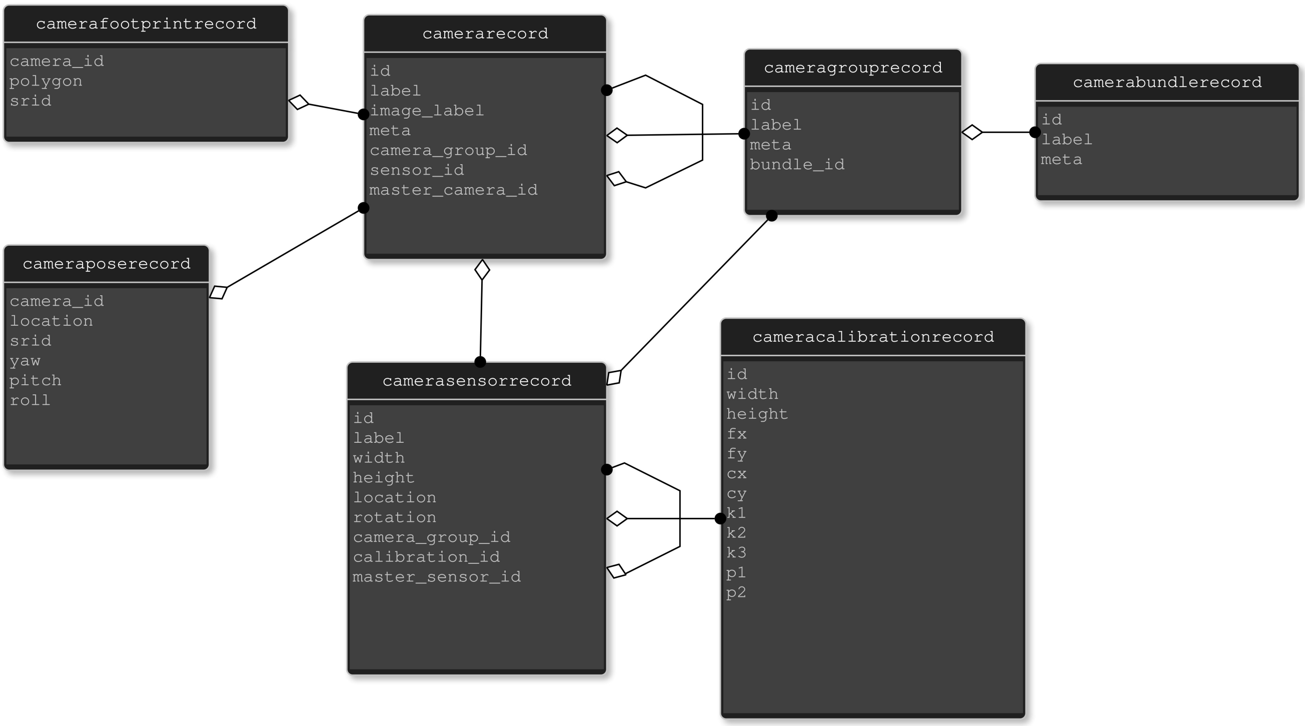

Figure S8. Entity relationship diagram (ERD) for the ORM models in the `sealoc.db` database in the SEALOC dataset. Models related by foreign keys are indicated by lines.
